# Targeted Dynamic Phospho-Proteogenomic Analysis of Gastric Cancer Cells Suggests Host Immunity Provides Survival Benefit

**DOI:** 10.1016/j.mcpro.2024.100870

**Published:** 2024-10-25

**Authors:** Kohei Kume, Midori Iida, Takeshi Iwaya, Akiko Yashima-Abo, Yuka Koizumi, Akari Endo, Kaitlin Wade, Hayato Hiraki, Valerie Calvert, Julia Wulfkuhle, Virginia Espina, Doris R. Siwak, Yiling Lu, Kazuhiro Takemoto, Yutaka Suzuki, Yasushi Sasaki, Takashi Tokino, Emanuel Petricoin, Lance A. Liotta, Gordon B. Mills, Satoshi S. Nishizuka

**Affiliations:** 1 Center of Molecular and Cellular Oncology, Yale University, New Haven, Connecticut, USA; 2 Department of Physics and Information Technology, Kyushu Institute of Technology, Iizuka, Fukuoka, Japan; 3 Department of Clinical Oncology, Iwate Medical University School of Medicine, Yahaba, Iwate, Japan; 4 Division of Biomedical Research & Development, Iwate Medical University Institute for Biomedical Sciences, Yahaba, Iwate, Japan; 5 Aspiring Scientists Summer Internship Program, Center for Applied Proteomics and Molecular Medicine, George Mason University, Manassas, Virginia, USA; 6 Center for Applied Proteomics and Molecular Medicine, George Mason University, Manassas, Virginia, USA; 7 Department of Genomic Medicine, University of Texas, MD Anderson Cancer Center, Houston, Texas, USA; 8 Department of Bioscience and Bioinformatics, Kyushu Institute of Technology, Iizuka, Fukuoka, Japan; 9 Department of Computational Biology and Medical Sciences, Graduate School of Frontier Sciences, The University of Tokyo, Kashiwa, Chiba, Japan; 10 Department of Medical Genome Sciences, Research Institute for Frontier Medicine, Sapporo Medical University, Sapporo, Hokkaido, Japan; 11 Knight Cancer Institute, Oregon Health and Science University, Portland, Oregon, USA

**Keywords:** 5-fluorouracil, cisplatin, copy number variation, gastric cancer, NFκB, PD-L1, proteogenomics, STAT1

## Abstract

Despite of massive emergence of molecular targeting drugs, the mainstay of advanced gastric cancer (GC) therapy is DNA-damaging drugs. Using a reverse-phase protein array-based proteogenomic analysis of a panel of 8 GC cell lines, we identified genetic alterations and signaling pathways, potentially associated with resistance to DNA-damaging drugs, including 5-fluorouracil (5FU), cisplatin, and etoposide. Resistance to cisplatin and etoposide, but not 5FU, was negatively associated with global copy number loss, vimentin expression, and caspase activity, which are considered hallmarks of previously established EMT subtype. The segregation of 19,392 protein expression time courses by sensitive and resistant cell lines for the drugs tested revealed that 5FU-resistant cell lines had lower changes in global protein dynamics, suggesting their robust protein level regulation, than their sensitive counterparts, whereas the cell lines that are resistant to other drugs showed increased protein dynamics in response to each drug. Despite faint global protein dynamics, 5FU-resistant cell lines showed increased signal transducer and activator of transcription 1 phosphorylation and PD-L1 expression in response to 5FU. In publicly available cohort data, expression of signal transducer and activator of transcription 1 and NFκB target genes induced by proinflammatory cytokines was associated with prolonged survival in GC. In our validation cohort, total lymphocyte count, rather than PD-L1 positivity, predicted a better relapse-free survival rate in GC patients with 5FU-based adjuvant chemotherapy than those with surgery alone. Moreover, total lymphocyte count^+^ patients who had no survival benefit from adjuvant chemotherapy were discriminated by expression of IκBα, a potent negative regulator of NFκB. Collectively, our results suggest that 5FU resistance observed in cell lines may be overcome by host immunity or by combination therapy with immune checkpoint blockade.

## Highlights

• Phospho-proteogenomic landscape of gastric cancer (GC) cells are described.
• Cisplatin-sensitive GC cells predominantly demonstrate chromosomal instability.
• Five-fluorouracil elicits PD-L1 expression in 5-fluorouracil–resistant GC cells.
• High expression of PD-L1 and NFκB target genes reflects host immunity.
• IκBα identifies patients who receive no survival benefit from chemotherapy.

## Graphical Abstract

**Figure:**
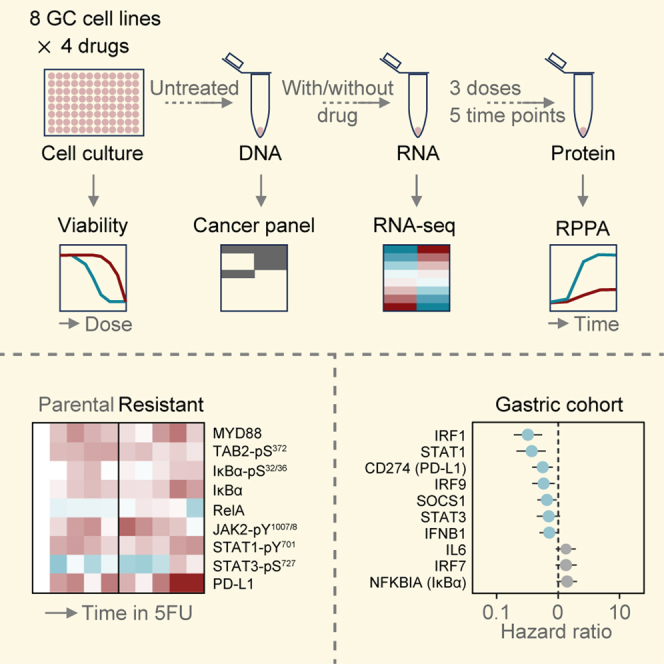

## In Brief

Kume *et al.* investigated signaling pathways linked to drug resistance across multiple gastric cancer (GC) cell lines using a dynamic phospho-proteogenomic strategy. In 5-fluorouracil-resistant lines, STAT1 phosphorylation and PD-L1 expression increased in response to 5-fluorouracil. Elevated levels of *STAT1*, *PD-L1*, and several proinflammatory gene expression were associated with prolonged survival in patients with GC, likely reflecting enhanced host immune response. These findings underscore the therapeutic potential of combination therapy with 5-fluorouracil and immune checkpoint blockade for GC patients.

Advanced gastric cancer (GC) is a leading cause of cancer death worldwide and there are limited treatment strategies for patients with this cancer (1). Multidisciplinary treatments, including combinations of surgery, chemotherapy, and radiotherapy have been used to improve survival for patients with GC (2). Indeed, curative surgery followed by adjuvant chemotherapy with an oral fluoropyrimidine S-1 containing the 5-fluorouracil (5FU) prodrug tegafur (3) decreases relapse and extends disease-free survival, particularly in the Japanese population (4, 5, 6). Unfortunately, a substantial number of patients still experience relapse. In the Japanese population, most relapses occur after S-1 adjuvant chemotherapy, suggesting that acquired 5FU resistance likely plays a substantial role for relapse. Clarifying the mechanisms leading to 5FU resistance offers the opportunity to select molecular targeting drugs designed to prevent relapse after treatment with DNA-damaging drugs.

Most GCs arise from glandular epithelia of the gastric mucosa that is exposed to a range of substances and stimulants that can promote host immune and inflammatory responses. Excessive activation of nuclear factor-κB (NFκB), which is considered to be a hallmark of inflammation-associated cancers (7), has been demonstrated to play a crucial role in GC progression and relapse (8, 9, 10). NFκB is activated not only by cytokines but also DNA-damaging drugs *via* the cytosolic DNA-sensing pathway (11). Stimulator of interferon genes (STINGs) is a key component of this DNA-sensing pathway and engages an inhibitor of nuclear factor-κB (IκB) kinase complex that phosphorylates IκB proteins to target them for proteasomal degradation. This degradation of IκB proteins allows NFκB/Rel transcription factors to enter the nucleus with subsequent activation of genes related to proinflammatory signaling. Despite the strong rationale for this pathway, there is limited information concerning the functional consequences of NFκB-mediated proinflammatory signaling in the response of GCs to 5FU.

Cancer cell lines are important model systems to study quantitative cellular and molecular response to external stimuli and drug uptake. 5FU-resistant GC cell lines have been used to demonstrate specific roles for 5FU metabolism, prostaglandin production, and autophagy in 5FU resistance (12, 13, 14). The cell lines MKN45/5FU (45FU) and MKN74/5FU (74FU) were established from the poorly differentiated MKN45 and differentiated MKN74 cell lines, respectively, by culturing in the presence of increasing concentrations of 5FU over the course of 1 year (15). Similar to previous work on tissue samples from 5FU-nonresponders (16), these cell lines showed increased expression of *thymidylate synthase* and decreased expression of *orotate phosphoribosyltransferase* compared to their parental counterparts. However, comprehensive knowledge of their genomic, transcriptomic, and proteomic profiles associated with phenotypic drug response to GC treatment is limited. Although the Cancer Genome Atlas and the Asian Cancer Research Group provide comprehensive genomic and transcriptomic profiles of GCs and further established a robust GC molecular classification method (17, 18), phenotypic drug responses, and proteomic profiles have not yet been integrated.

In the present study, we used our dynamic phosphoproteomics platform, reverse-phase protein array (RPPA), with GC cell lines to determine the association between key molecules that have been validated in real-world cohort data and the host immune responses. Our findings suggest that the mechanisms of 5FU resistance observed in cell line models can be counteracted by lymphocyte-mediated host immunity potentially in the presence of immune checkpoint blockade.

## Experimental Procedures

### Cell Culture

GCIY, GSS, MKN1, MKN45, and MKN74 cells were obtained from the RIKEN Cell Bank. 5FU-resistant lines 45FU and 74FU were established from MKN45 and MKN74 cells, respectively, as described previously (15). IWT-1 is a cell line that was established in our laboratory from a male Japanese patient with GC who had relapsed with peritonitis carcinomatosa (19). Use of the IWT-1 cell line was approved by the Iwate Medical University Institutional Review Board (IRB, H25-116, and HG H25-15), and written informed consent was obtained from the family of donor patient, who had died at the time the cell line was established. The IWT-1 cell line is now distributed *via* the RIKEN BRC Cell Bank (https://cell.brc.riken.jp/en/). All 8 cell lines were grown in RPMI 1640 media (Life Technologies), supplemented with 10% fetal bovine serum (Life Technologies), and cultured at 37 °C in a humidified incubator supplied with 5% CO_2_.

### Reverse-Phase Protein Array

For sample collection, cells were exposed to anticancer drugs (5FU, cisplatin [CIS], etoposide [ETP], and docetaxel [DTX]) in a 96-well plate at low, medium, and high concentrations (Supplemental Table S1). Each concentration was applied at five time points over 24 h. Cells were collected by trypsinization followed by pipetting and centrifugation at 1700*g* for 2 min at 4 °C. The resulting cell pellet was stored at −80 °C until further RPPA analysis as previously described (20). For RPPA assays, cell pellets were processed to obtain cell lysates according to previously published protocols (20). An individual RPPA slide contains 2000 dots for 12 conditions × 5 time points × 8 cell lines × 4 replicates, and 80 dots for control MIX samples and buffer control (21, 22). The dots were printed on nitrocellulose-embedded glass slides (Grace BioLabs) with an Aushon 2470 Microarrayer (Quanterix). The Mix sample contains a variety of sample lysates from all possible drug administration conditions. Each sample lysate was spotted in tetraplicate for quantitative analysis and subsequently probed with individual primary antibodies (Supplemental Table S2) for which the specificity was verified by strip Western blotting (21, 22). Phosphoproteins assayed are annotated in Supplemental Table S2. A total of 19,392 protein expression time courses shown in Figure 3*F* were analyzed by staining 96 time-course lysate series, each with 202 antibodies. To block nonspecific interactions, SuperG (Grace Biolabs), iBlock (Life Technologies), or standard bovine serum albumin solutions were used depending on the antibody. Signals were obtained using a tyramide signal amplification kit (Thermo Fisher Scientific) and subsequently quantified using a TissueScope 4000 Scanner (Huron Technologies).

**Fig. 1:**
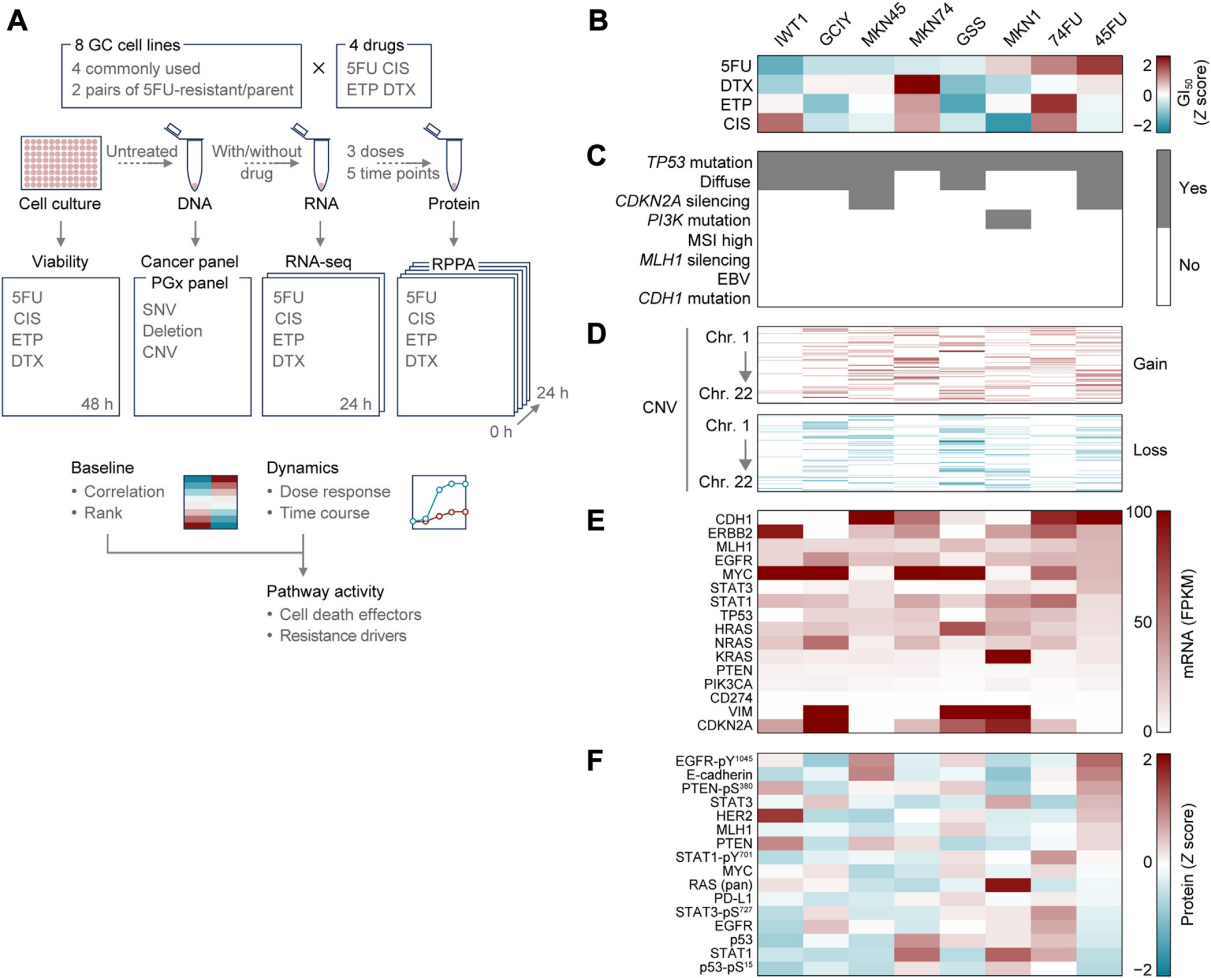
**Integrative multiplatform analyses of GC cell lines.***A*, experimental outline. A panel of 8 GC cell lines were analyzed using multiple platforms including: (i) water-soluble tetrazolium salt-based cell viability assay; (ii) a large panel-based targeted gene sequencing; (iii) RNA sequencing (RNA-seq); and (iv) reverse-phase protein array (RPPA). Sensitivity to each drug was evaluated on the basis of 50% growth inhibitory concentration (GI_50_). Pathway activity was determined based on the fold changes in mRNA and protein levels from baseline to 24 h after drug treatment. 5FU, 5-fluorouracil; CIS, cisplatin; ETP, etoposide; DTX, Docetaxel; PGx, pharmacogenomics. *B–F*, GI_50_ for each drug tested (*B*), cell line summary (*C*), copy number variation (CNV) landscape (*D*), and mRNA and protein expression at baseline (*E* and *F*) are depicted. Cell lines are ordered by 5FU sensitivity. DTX was used as a non-DNA-damaging drug. Relevant genes for cell line summary and expression profiles were selected based on previous studies of GC subtypes (17, 18). EBV gene expression was detected by quantitative RT-PCR (Supplemental Fig. S1*C*).

**Fig. 2:**
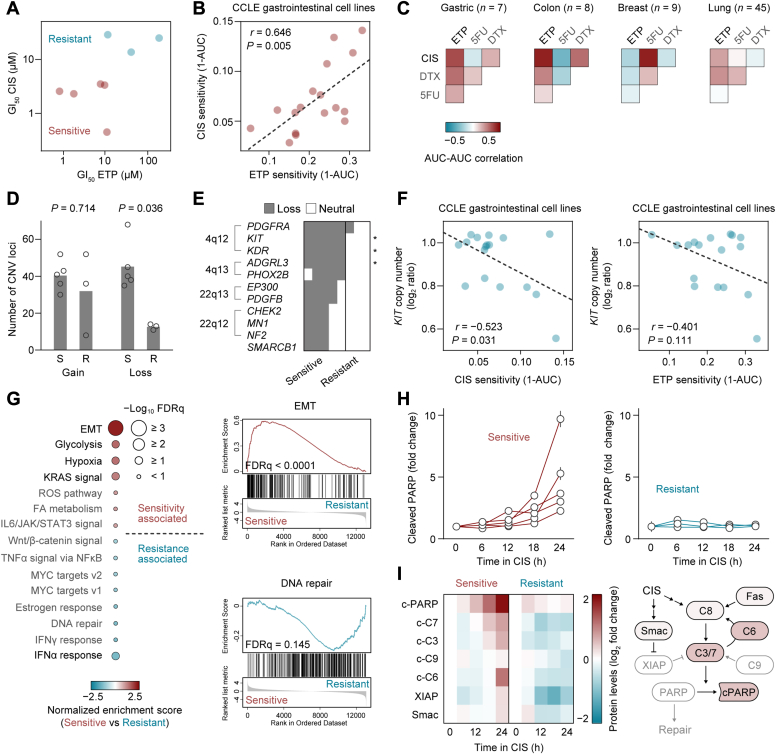
**CNV and mesenchymal gene expression in CIS-sensitive GC cells.***A*, GI_50_ profiles of GC cell lines to define sensitivity and resistance to CIS and ETP. *B*, scatter plot showing correlation between area under the dose-response curve (AUC) values for CIS and ETP across gastric and colon cancer cell lines. *Each dot* indicates an individual cell line (*n* = 17) shared between the AUC data sets. *C*, heatmaps represent pairwise Pearson correlations between AUC values of CIS and ETP assessed in the PRISM repurposing secondary screen (30). *D*, number of CNV loci detected. *Each dot* indicates an individual cell line. The two-tailed *p* values were obtained with a nonparametric Mann-Whitney *U* test. *E*, binary matrix representing genes that commonly lost its copy number in cell lines with dual CIS/ETP sensitivity. The two-tailed *p* values were obtained with Fisher’s exact test. *F*, scatter plot showing correlation between AUC values of CIS or ETP and *KIT* copy number across gastric and colon cancer cell lines. *Each dot* indicates an individual cell line. *G*, hallmark GSEA signatures from RNA-seq data ranked by Normalized Enrichment Score (NES) for CIS/ETP-sensitive *versus* CIS/ETP-resistant cell lines (*left*). GSEA plots showing positive and negative enrichment of “EMT” and “DNA repair” gene sets in CIS/ETP-sensitive cell lines (*right*). FDRq, false discovery rate (*q* value). *H*, time course RPPA data showing changes in cleaved PARP levels after CIS treatment. Error bars represent s.e.m. *I*, caspase-focused RPPA analysis of dual CIS/ETP-sensitive (mean, *n* = 5) and -resistant (mean, *n* = 3) cell lines (*left*) and a schematic of CIS-activated signaling outcomes in dual CIS/ETP-sensitive cell lines (*right*). c-C3−9, cleaved caspase-3−9; c-PARP, cleaved PARP.

**Fig. 3:**
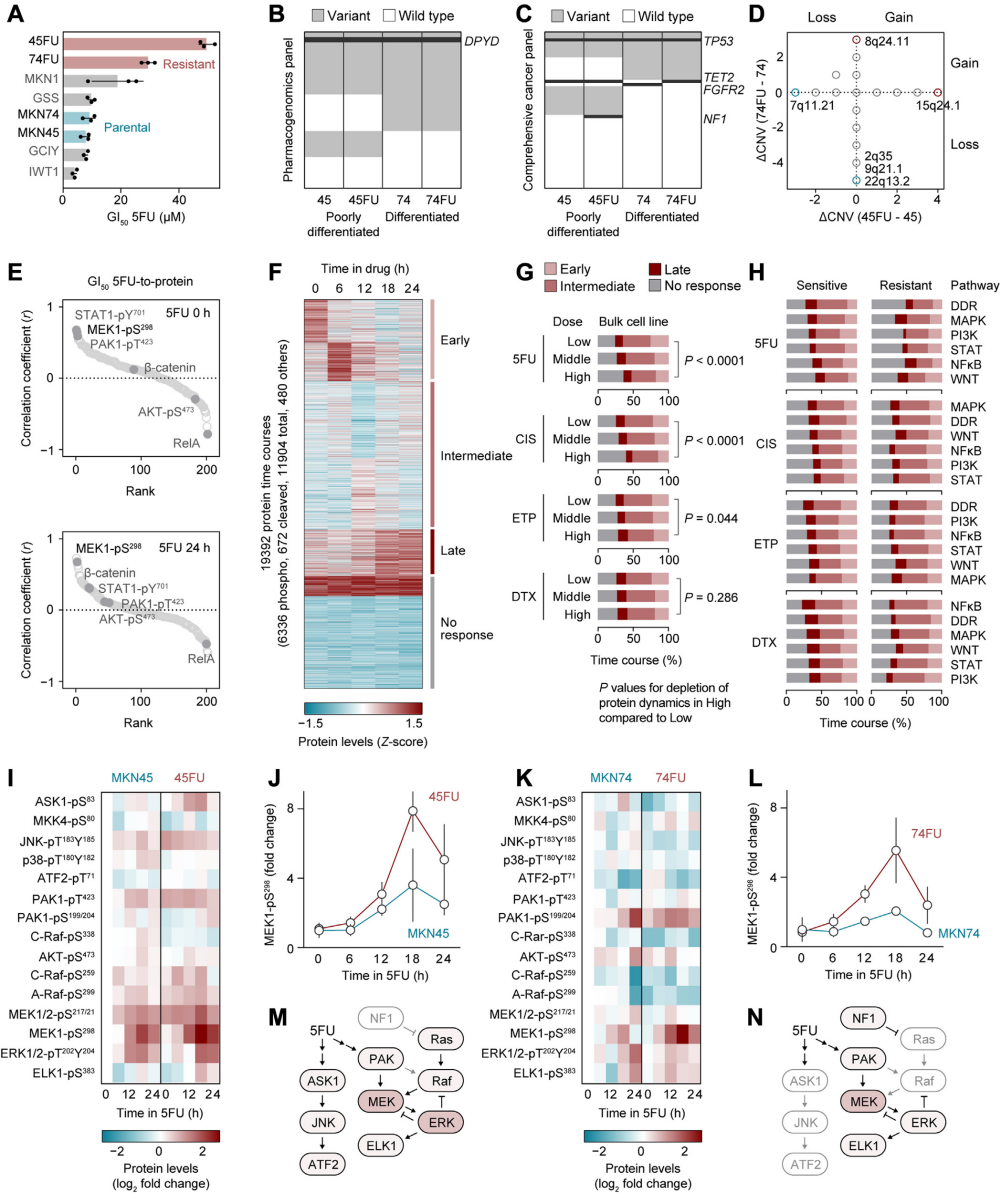
**5FU-induced MAPK signaling pathways.***A*, GI_50_ profiles of GC cell lines to define 5FU sensitivity and resistance. Two pairs of 5FU resistant and parental cell lines are highlighted. *B*, pharmacogenomics panel shows drug metabolizing genes harboring single-nucleotide variants (SNVs) in each cell line. *DPYD* is highlighted as a canonical 5FU metabolism pathway gene. *C*, comprehensive cancer panel. *TP53*, *TET2*, *FGFR2*, and *NF1* are highlighted for common, MKN74-unique, and 45FU-unique mutations. *D*, CNV analysis showing no shared copy number changes between 74FU and 45FU. Copy number changes were calculated by subtracting the copy number of each chromosomal position in matched parental cell lines from that in resistant cell lines (ΔCNV). Chromosomal positions with maximum and minimum ΔCNVs are highlighted. *E*, Pearson correlations between GI_50_ 5FU and protein expression at baseline (*top panel*) and 24 h after 5FU treatment (*bottom panel*). *F*, temporal proteomic changes in 8 cell lines within 24 h of 5FU, CIS, ETP, or DTX treatment. Seven clusters were determined by K-means clustering and further grouped for early, intermediate, late, and no response based on their kinetics. *G*, proportions of protein expression time courses segregated into three doses for each drug. Two-tailed *p* values were obtained with Fisher’s exact test. *H*, proportions of protein expression time courses from high-dose conditions segregated into six signaling pathways including DNA damage response (DDR), MAPK, PI3K, STAT, NFκB, and WNT pathways. *I*, MAPK pathway-focused RPPA analysis of 45FU and parental MKN45 cells. *J*, temporal changes in MEK1-pS^298^ levels after 5FU treatment. *K*, MAPK pathway-focused RPPA analysis of 74FU and parental MKN74 cells. *L*, temporal changes in MEK1-pS^298^ levels after 5FU treatment. *M*, 5FU-activated MAPK signaling pathways in 45FU cells. *N*, 5FU-activated MAPK signaling pathways in 74FU cells. Error bars represent s.d. (*A*) or s.e.m. (*J* and *L*).

### Cohorts (Discovery and Validation)

GSE62254 dataset (*n* = 300) was used to determine proinflammatory cytokine-responsive genes that are associated with survival of GC patients. The Northern Japan Gastric Cancer Study patient cohort (NCT01905969, *n* = 658) was used to examine the effects of total lymphocyte count (TLC), tumor PD-L1 and IκBα expression, and *Helicobacter pylori* positivity on treatment outcomes in advanced GC patients. The multicenter study protocol was approved by the IRB of Iwate Medical University (H24-132, HGH24-12, and HG2020-018), where all analysis was carried out. Ethical approval for conducting this study was given by all IRB committees of all 12 participating institutions listed in the Northern Japan Gastric Cancer Study Consortium (23, 24, 25). The analysis with this cohort was conducted in accordance with the Declaration of Helsinki.

### Experimental Design and Statistical Rationale

For multiplatform analysis of genetic alteration, gene expression, and protein phosphorylation, which potentially associated with drug resistance, we collected DNA, RNA, and whole-cell lysates from 8 GC cell lines including two pairs of 5FU-resistant cell lines and their parental counterparts. Cell viability assay was used to validate 5FU resistance of the 5FU-resistant cell lines and determine the cell lines that were resistant to other drugs. We took advantage of RPPA to analyze thousands of samples simultaneously, which is useful for time course experiments with many cell types. Cell cultures were scale down from the commonly used 25 cm^2^ flasks to 96-well plates to minimize sample handling by using a multichannel pipette. Key molecules associated with 5FU resistance were validated in real-world cohort data.

Statistical analysis was performed using GraphPad Prism (GraphPad Software, www.graphpad.com). Statistical significance of differences between two groups was evaluated using an unpaired two-tailed *t* test, or as indicated in the figure legends. For the correlation analysis, Pearson correlation coefficient *r* and *p* value in linear regression were used to evaluate the strength and statistical significance of the correlation. Differences having a *p* value of < 0.05 were considered statistically significant for screening purposes. For heat map visualization, genes or proteins were ordered by average-linkage clustering, complete-linkage clustering, or from highest to lowest values in a column, unless otherwise stated. For survival analysis, patients were divided into two groups based on the median lymphocyte count or immunohistochemistry positivity.

## Results

### Proteogenomic Profiles of GC Cell Lines with Phenotypic Drug Response

We started by characterizing GC cell lines in terms of fifty percent growth inhibitory concentration (GI_50_) for different chemotherapeutic agents, GC-associated mutations (17, 18, 26), and RNA and protein expression profiles (Fig. 1*A*). Although 5FU resistance was observed for both 45FU and 74FU cell lines (15), 74FU also had CIS and ETP resistance, indicating cross resistance among DNA-damaging drugs (Fig. 1*B*; Supplemental Fig. S1, *A* and *B*). Neither cell line showed apparent resistance to the non–DNA-damaging drug DTX that function as a mitotic inhibitor. As expected from gene alterations reported for GC, all cell lines tested carried *TP53* mutations (Fig. 1*C*). The cell line MKN1 carried a *PIK3CA* mutation, one of the key determinants of the Epstein–Barr virus–positive GC subtype (Fig. 1*C*), but none of the 8 cell lines tested showed Epstein–Barr virus gene expression (Supplemental Fig. S1*C*). Copy number variation (CNV) analysis revealed a positive correlation between the number of CNV loci and CIS sensitivity (Fig. 1*D*). This correlation resembles CIS hypersensitivity of cells derived from Fanconi anemia patients as a phenotypic consequence of genomic instability (27). CIS sensitivity was also associated with a lack of vimentin silencing (28, 29) (Fig. 1*E*). Most GC-associated proteins tested were correlated with their corresponding mRNA levels, except for MYC and phosphoproteins such as STAT3-pS^727^ and EGFR-pY^1045^ (Fig. 1*F*; Supplemental Fig. S1*E*). As reflected by their differential drug responses, 45FU and 74FU do not share similarly expressed genes and proteins, except for *CDH1*, which encodes E-cadherin (Fig. 1*F*). Indeed, both lines retained most of the genetic programs of their parental cells (Supplemental Fig. S1*D*). Thus, we further examined reasons for the correlation between CIS sensitivity and increased number of CNV loci, and then pursued exploratory RPPA analysis to identify shared signaling outcomes between 45FU and 74FU.

### CIS-Sensitive GC Cells Predominantly Demonstrate CIN

To examine reasons for the correlation between CIS sensitivity and increased number of CNV loci, we segregated CIS-sensitive and CIS-resistant cell lines based on their GI_50_ CIS profiles (Fig. 2*A*). As expected from Figure 1*B*, the same cell lines showed CIS and ETP sensitivity and resistance. This observation is further supported by the PRISM repurposing primary screen (30), showing a positive correlation between CIS and ETP sensitivity for both gastric and colon cancer cell lines (Fig. 2, *B* and *C*; Supplemental Table S3). Although no difference in CNV gain is observed, a 3-fold higher level of CNV loss was detected in dual CIS/ETP-sensitive cell lines than their resistant counterparts (Fig. 2*D*). Genes that were frequently lost included *c-kit* (*KIT*), *kinase insert domain–containing receptor* (*VEGFR2*), and *adhesion G protein-coupled receptor L3*, which are all located on chromosome at 4q12–q13 (Fig. 2*E*). Validation with whole genome data of the PRISM GC cell lines confirmed negative correlation between CIS/ETP sensitivity and *KIT* copy number (Fig. 2*F*), and further identified several gains and losses correlated with CIS sensitivity, such as proximal 1p32-33 gains (*CDKN2C*, *CMPK1*, and *TAL1*) and 20q13 losses (*GNAS*, *AURKA*, *TOP1*, *PLCG1*, *MAFB*, and *PTPRT*) (Supplemental Fig. S2*A*). Among the top three candidates for both gains and losses, *CDKN2C* gain, *KIT* loss, and *GNAS* loss were unique to GC cell lines, while *ERBB3* gain, *FBXW* gain, and hypoxia-inducible factor 1a loss were also observed in other cancer types (Supplemental Fig. S2*B*). Thus, CIS sensitivity in GC cell lines could be defined by chromosomal instability (CIN), represented by loss of proximal 4q12–q13, which could be related to the *KIT* copy number.

### Lack of Vimentin Silencing is Associated with CIS/ETP Sensitivity Induced by CIN

We next investigated the positive correlation between CIS sensitivity and the lack of vimentin silencing. As expected, gene set enrichment analysis (GSEA) showed that “epithelial-mesenchymal transition (EMT)” was the most enriched upregulated gene set in cell lines having dual CIS/ETP sensitivity (Fig. 2*I*, left). RNA-seq gene expression and mass spectrometry–based proteomic data from the Cancer Cell Line Encyclopedia also showed a positive correlation between both vimentin gene and protein expression with CIS sensitivity across gastric and colon cancer cell lines (Supplemental Fig. S2, *A* and *B*), which complements our RPPA-based proteomic data. TWIST, one of the master transcription factors of the vimentin gene, is directly regulated by hypoxia-inducible factor 1a, a key regulator of glycolytic genes, and promotes metastasis (31). Supporting this transcriptional mechanism, “glycolysis” and “hypoxia” genes are concurrently enriched for cell lines having dual CIS/ETP sensitivity (Fig. 2*I*, left). These cell lines also showed negative enrichment of “interferon (IFN)α response” and “IFNγ response” pathways. Interestingly, recent work showed that CIN-induced chronic activation of cyclic GMP–AMP synthase-STING, which is a driver of cancer metastasis, led to STING depletion, thereby reducing IFN responsiveness (32).

We also explored central effectors of cell death pathways that are activated by CIS and ETP in GC cells. Consistent with the correlation between CIS/ETP sensitivity and CIN, downregulated genes are significantly enriched for the “DNA repair” pathway (Fig. 2*G*). Pairwise comparisons of time course protein expression profiles between dual CIS/ETP-sensitive and CIS/ETP-resistant cell lines revealed consistently low levels of cleaved PARP in dual CIS/ETP-resistant cell lines regardless of CIS or ETP treatments (Fig. 2*H*; Supplemental Fig. S2*C*). Subsequent caspase-focused analysis highlighted lack of cleaved caspase-3, caspase-7, and caspase-6 in dual CIS/ETP-resistant cell lines following CIS treatment (Fig. 2*I*). Both cleaved caspase-3 and caspase-7 cleave PARP to inhibit its enzymatic activity (33), whereas cleaved caspase-6 creates a positive feedback loop with caspase-8, caspase-7, and caspase-3 (34, 35). Therefore, caspase-6 cleavage appears to be a key event in the CIS response in GC cells. Meanwhile, levels of cleaved caspase-9 and X-linked inhibitor of apoptosis protein concurrently increased in response to ETP treatment, reflecting the early plateau of PARP cleavage (supplemental Fig. S2*D*).

Taken together, the lack of vimentin silencing can impose a fitness cost on dual CIS/ETP-sensitive cell lines, resulting in increased metabolic requirements and a lower threshold for caspase activation.

### 5FU-Induced MAPK Signaling Pathways Converge on Phosphorylation of MEK1

Next, we pursued proteogenomic analysis to identify shared signaling outcomes in the two 5FU-resistant cell lines, 45FU and 74FU (Fig. 3*A*). Pairwise comparison of targeted pharmacogenomic and comprehensive cancer panel sequencing-based mutation profiles between 5FU-resistant and matched parental cell lines identified a missense mutation in *neurofibromatosis type 1* (*NF1*), c.5461G>T (V1821F) that is unique to the 45FU cell line (Fig. 3, *B* and *C*). *NF1* encodes a GTPase-activating protein that negatively regulates Ras pathway activity by accelerating the hydrolysis of Ras-bound GTP, thereby acting as a tumor suppressor (36). However, *NF1*^V1821F^ is not listed in the Genome Aggregation Database (gnomAD) (https://gnomad.broadinstitute.org/) as a pathogenic mutation. Previous studies showed that a missense mutation in *dihydropyrimidine dehydrogenase* (*DPYD*), c.85T>C (C29R), confers gain-of-function activity (37, 38). Despite the 3- to 5-fold difference in 5FU sensitivity between the 5FU-resistant and matched parental cell lines, both pairs harbor the same *DPYD*^C29R^ mutation. Consistent with previous reports (15, 16), both 5FU-resistant cell lines showed a 2- to 4-fold increase in *thymidylate synthase* expression compared to their matched parental cell lines in the presence of 5FU, suggesting that enhanced 5FU metabolism contributes in part to the phenotypic 5FU response (Supplemental Fig. S3*A*). Although we detected no unique mutations in 74FU cells, the parental cell line MKN74 has a unique mutation in *fibroblast growth factor receptor 2*, c.1199G>A (R400Q), suggesting that this cell line contains a minor subpopulation with WT *fibroblast growth factor receptor 2* that confers a survival advantage in the presence of 5FU. In contrast, 74FU cells harbor a missense mutation in *Tet methylcytosine dioxygenase*, c.367C>T (R123C), which was not detected in the parental MKN74 cells. Unlike dual CIS/ETP sensitivity determined by the loss of proximal 4q12–q13, pairwise comparison of CNV profiles between the 5FU-resistant and matched parental cell lines identified no copy number changes shared by 45FU and 74FU cell lines (Fig. 3*D*). To identify proteins associated with 5FU resistance, we thus determined the correlation between protein level and GI_50_ 5FU using Pearson correlation coefficients (Fig. 3*E*). At baseline, signal transducer and activator of transcription (STAT1)-pY^701^, MAP-extracellular signal–regulated kinase (MEK) 1-pS^298^, and Rac1 p21-activated kinase (PAK) 1-pT^423^ are the top three proteins that positively correlated with GI_50_ 5FU. At 24 h after 5FU treatment, MEK1-pS^298^, but not STAT1-pY^701^ and PAK1-pT^423^, remained as highly expressed, suggesting that MEK1-pS^298^ is a key driver molecule of 5FU resistance.

### Dynamic Phosphoproteomics Identifies MEK1 as a Key 5FU-Resistance Pathway Molecule

To see how 5FU affects signaling pathway activity in 5FU-resistant and 5FU-sensitive cell lines, we divided the protein expression time courses into four groups, early (peaked by 6 h), intermediate (peaked by 12 h), late (peaked by 24 h), and no response (flat), and then determined the proportions of these dynamics in different signaling pathways including DNA damage response (DDR), mitogen-activated protein kinase (MAPK), PI3K, STAT, NFκB, and WNT (Fig. 3, *F*–*H*). Compared to a non–DNA-damaging drug DTX, DNA-damaging drugs including 5FU, CIS, and ETP reduced the proportions of early, intermediate, and late protein changes in a dose-dependent manner, indicating depletion of protein dynamics (Fig. 3*G*). The segregation of protein expression time courses by sensitive and resistant cell lines revealed that 5FU-resistant cell lines had lower changes in global protein expression dynamics than their sensitive counterparts, whereas the cell lines that are resistant to other drugs showed increased protein expression dynamics in response to each drug (Fig. 3*H*). Among the signaling pathways tested, the MAPK pathway appears to remain active in the 5FU-resistant cells having global protein dynamics depletion (Fig. 3*H*), which is consistent with the positive correlation between MEK1-pS^298^ levels and GI_50_ 5FU (Fig. 3*E*).

To identify MAPK signaling outcomes that are shared between 45FU and 74FU, we examined a phosphoproteomic analysis involving RPPA that was focused on the MAPK pathway. Among the three major MAPKs, c-Jun N-terminal kinase (JNK), p38, and extracellular signal–regulated kinase (ERK), JNK and p38 are activated by apoptosis signal–regulating kinase 1 (ASK1) in response to a diverse array of stresses such as oxidative stress, endoplasmic reticulum stress, and calcium influx (39), but no established stress-responsive upstream kinases have been reported for ERK. Interestingly, a previous study suggested that Rac1 GTPase, a PAK activator, plays an essential role in activation of gamma-irradiation–induced ERK1/2 signaling in the breast cancer cell line MCF7 (40). PAK has been demonstrated to phosphorylate S298 of MEK1 and S338 of C-Raf (41, 42). Therefore, we included PAK as an upstream kinase of Raf and MEK that can respond to 5FU. As expected from the genetic profiles, the effects of 5FU on signaling pathways differed between 45FU and 74FU cells (Fig. 3, *I–L*). The 45FU cells showed higher amplitude of ASK1 phosphorylation following 5FU treatment than did the parental cells, whereas phosphorylation levels of both ASK1 and its downstream kinase JNK were largely similar in both 74FU and its parental cell line (Fig. 3, *I* and *K*). In addition to ASK1, phosphorylation of A-Raf at S299 also increased in 45FU cells in response to 5FU, with baseline activity also being higher than in parental cells, potentially due to the loss-of-function *NF1* mutation in the cell line (Fig. 3*C*). Meanwhile, phosphorylation of C-Raf at S338 was instead depleted in 45FU cells, along with an increase in inhibitory phosphorylation at S259 (Fig. 3*I*). For 74FU cells, smaller or no changes in A-Raf phosphorylation were seen and only phosphorylation of PAK being observed among kinases upstream of MEK (Fig. 3*K*). Notably, both 45FU and 74FU cells showed a sharp increase in MEK1 phosphorylation at S298 in response to 5FU (Fig. 3, *J* and *L*). However, phosphorylation of the downstream kinase ERK did not reflect the amplitude and timing of MEK1-pS^298^ peaks (Supplemental Fig. S3, *B* and *C*). Thus, regardless of their distinct genomic alterations, 5FU-induced MAPK signaling pathways in 5FU-resistant cells converges on phosphorylation of MEK1 either *via* PAK alone or both PAK and A-Raf (Fig. 3, *M* and *N*).

### 5FU Stimulates Divergent Signaling Cascades in 5FU-Resistant GC Cells

**Fig. 4:**
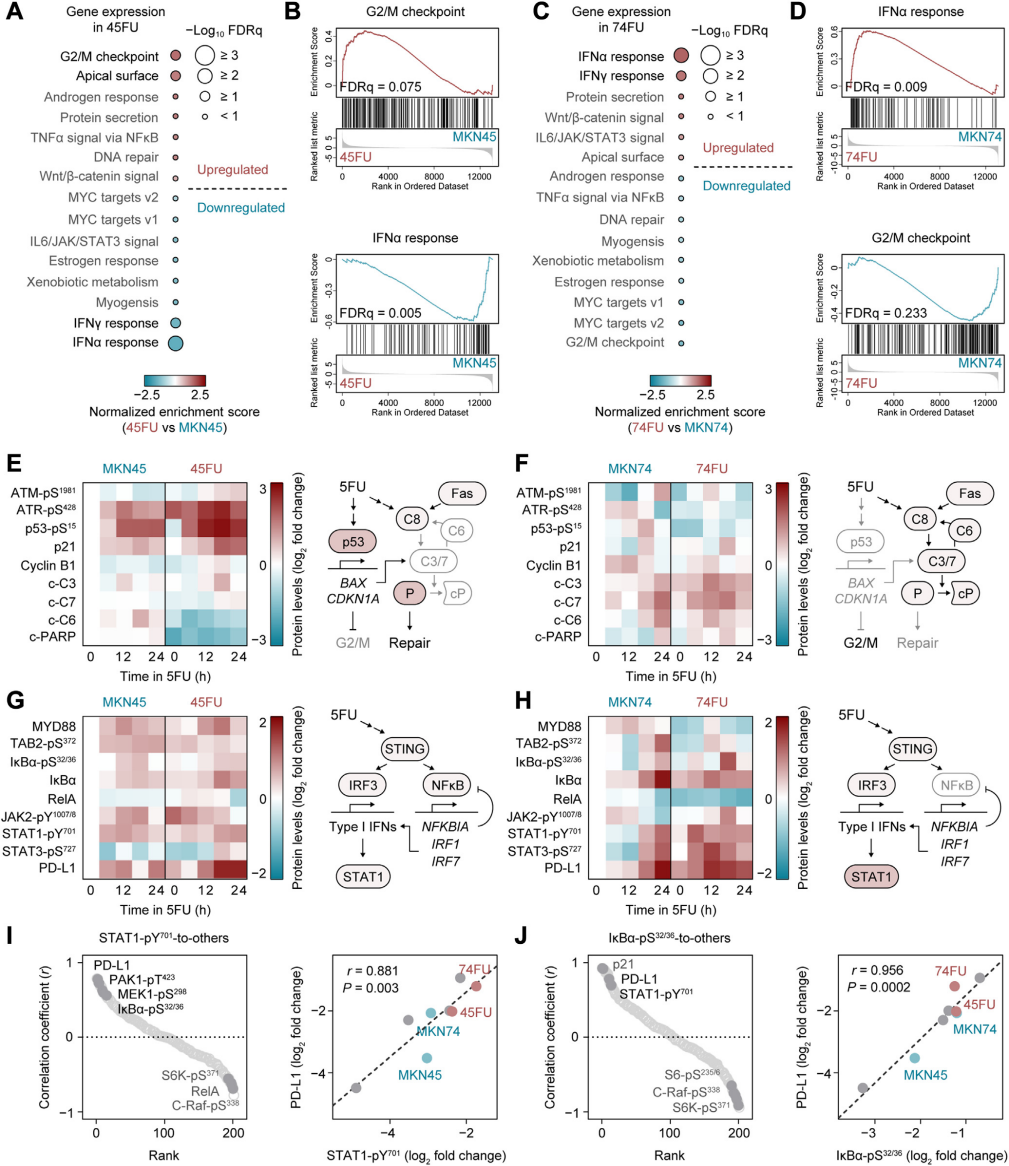
**5FU elicits PD-L1 expression in 5FU-resistant GC cells.***A*, hallmark GSEA signatures from RNA-seq data ranked by NES for 45FU *versus* parental MKN45 cells. *B*, GSEA plots showing positive and negative enrichment of “G2/M checkpoint” and “IFNα response” gene sets in 45FU cells. *C*, hallmark GSEA signatures from RNA-seq data ranked by NES for 74FU *versus* parental MKN74 cells. *D*, GSEA plots showing positive and negative enrichment of “IFNα response” and “G2/M checkpoint” gene sets in 74FU cells. *E*, DNA damage response (DDR) pathway-focused RPPA analysis of 45FU and parental MKN45 cells (*left*) and a schematic summarizing signaling outcomes in 45FU cells (*right*). *F*, DDR pathway-focused RPPA analysis of 74FU and parental MKN74 cells (*left*) and a schematic summarizing signaling outcomes in 74FU cells (*right*). c-PARP, cleaved PARP; c-C3−9, cleaved caspase-3−9. *G*, STAT and NFκB (proinflammatory) pathway-focused RPPA analysis of 45FU and parental MKN45 cells (*left*) and a schematic summarizing signaling outcomes in 45FU cells (*right*). *H*, proinflammatory pathway-focused RPPA analysis of 74FU and parental MKN74 cells (*left*) and a schematic summarizing signaling outcomes in 74FU cells (*right*). *I*, Pearson correlations between STAT1-pY^701^ expression and expression of other proteins (*left*) and a representative scatter plot showing correlation between STAT1-pY^701^ and PD-L1 (*right*). *J*, Pearson correlations between IκBα-pS^32/36^ expression and expression of other proteins (*left*) and a representative scatter plot showing correlation between IκBα-pS^32/36^ and PD-L1 (*right*). FDRq, false discovery rate (*q* value) (*B* and *D*).

To support the results of the RPPA-based analysis, we also used GSEA for pathway profiling of 45FU and 74FU cells (Fig. 4, *A–D*). Unexpectedly, “G2/M checkpoint” was the top upregulated pathway in 45FU cells, but was the top downregulated pathway in 74FU cells, and vice versa for “IFNα response” (Fig. 4, *A* and *C*). To visualize the distinct signaling outcomes, we analyzed RPPA datasets focused on DDR for activation of “G2/M checkpoint” and STAT for “IFNα response” (Fig. 4, *E–H*). Although the DDR pathway is known to be upregulated upon 5FU treatment, to our knowledge, established 5FU-responsive regulators in the STAT pathway have not been reported. The DNA sensing cyclic GMP–AMP synthase–STING pathway is known to activate IRF3 and NFκB in the presence of damaged DNA (11). Both IRF3 and transcriptional targets of NFκB like IRF1 and IRF7, can upregulate type I IFNs expression that in turn activates the STAT pathway. Thus, we included proteins and phosphoproteins in the NFκB pathway to assess potential IFNα responses. Consistent with the GSEA data, 45FU cells showed increased DDR pathway activity in response to 5FU, as indicated by higher amplitudes of ATR-pS^428^, p53-S^15^, and p21 expression than their parental cells, although levels of ATR-pS^428^ were high at baseline (Fig. 4*E*). Of note, 45FU cells are characterized by decrease in cleaved PARP levels, suggesting that intact PARP could detect DNA lesions and recruit repair proteins to the DNA damage (43). In contrast to 45FU cells, 74FU cells exhibit little or no changes in DDR pathway protein levels except for increased levels of cleaved caspases (Fig. 4*F*). As indicated by increased levels of myeloid differentiation factor 88, TAK1-binding protein 2-pS^372^ and IκBα-pS^32/36^, the NFκB pathway was activated by 5FU in 45FU cells (Fig. 4*G*). Meanwhile, 74FU cells display decreased levels of myeloid differentiation factor 88 and TAK1-binding protein 2-pS^372^ at baseline (Fig. 4*H*), and instead have higher amplitudes of STAT1-pY^701^ and STAT3-pS^727^ expression than 45FU cells (Fig. 4, *G* and *H*). This result is in good agreement with GSEA data showing “IFNα response” and “IFNγ response” among the most upregulated pathways (Fig. 4*C*).

### 5FU Specifically Elicits PD-L1 Expression *via* STAT1 Phosphorylation

Notably, both 45FU and 74FU cells demonstrated increased expression of programmed death ligand-1 (PD-L1) in response to 5FU (Fig. 4, *G* and *H*). A previous study reported that surface expression of PD-L1 and phosphorylation of STAT1 in pancreatic cancer cell lines was elicited by treatment with conventional chemotherapeutic drugs including 5FU, gemcitabine, and paclitaxel, although the correlation with phenotypic drug response was not clear (44). To clarify whether non–DNA-damaging drugs like paclitaxel and DTX have similar effects as DNA damaging drugs on IFN responses, we used GSEA to compare expression of IFN-responsive genes in GC cell lines with and without CIS, ETP, 5FU, or DTX treatment. Interestingly, expression of IFNα and IFNγ pathway genes was upregulated by treatment with CIS, ETP, or 5FU, but not with DTX, whereas NFκB pathway genes were upregulated by all drugs tested (Supplemental Fig. S4). To examine the significance of PD-L1 expression among the proteins that are coexpressed with STAT1-pY^701^, we determined the correlation between expression levels of STAT1-pY^701^ and the other proteins or phosphoproteins using Pearson correlation analyses (Fig. 4*I*). In line with the scenario, PD-L1 correlation is ranked on top along with PAK1-pT^423^/PAK2-pT^402^, MEK1-pS^298^, and IκBα-pS^32/36^. The correlation between expression levels of IκBα-pS^32/36^ and these other proteins further emphasized an association between STAT1 and PD-L1 (Fig. 4*J*). Collectively, these results suggest that 5FU elicits not only divergent signaling cascades in 5FU-resistant cells but also convergent PD-L1 expression.

### Lymphocyte Count Predicts a Survival Benefit with Adjuvant Chemotherapy

PD-L1, also known as B7-H1 or CD274, is a ligand for PD-1 that inhibits T cell activation by binding to PD-1 on T cells (45). Anti-PD-L1/PD-1 antibodies together with an S-1 adjuvant chemotherapy containing the 5FU prodrug tegafur (3), has become a standard treatment for locally advanced GC in Asia (46). Based on the observation that the two 5FU-resistant cell lines showed increased levels of PD-L1 compared to their parental cell lines, we examined whether PD-L1 could be a prognostic factor for GC patients who underwent gastrectomy followed by S-1 adjuvant chemotherapy using the Northern Japan Gastric Cancer Study patient cohort (23, 24, 25).

**Fig. 5:**
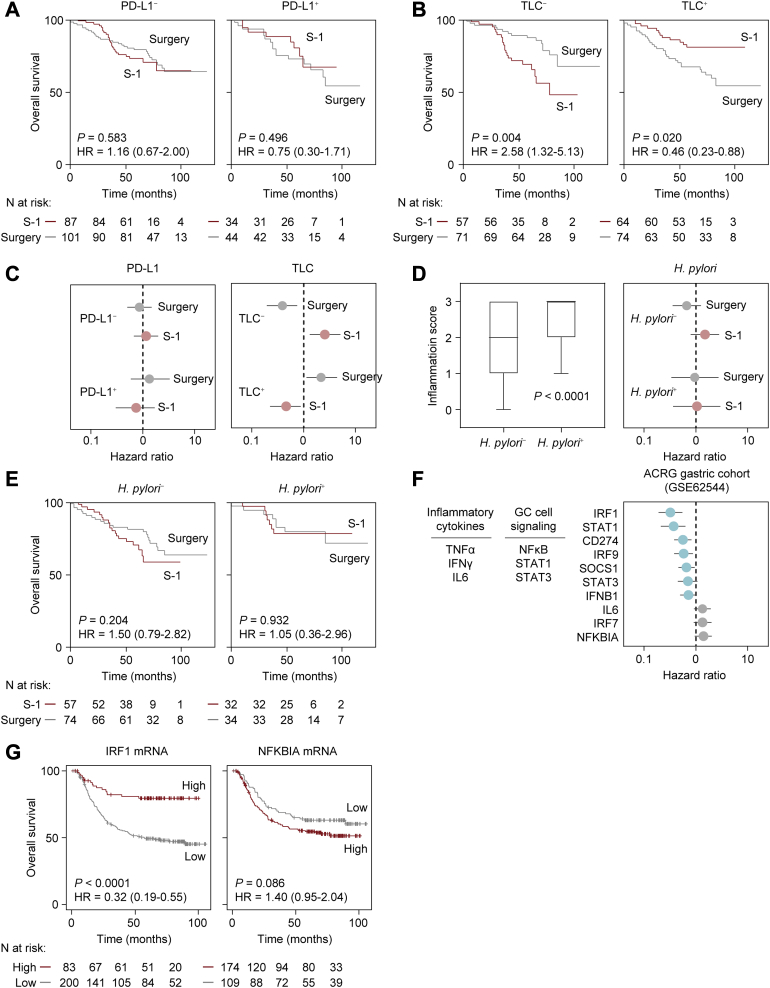
**Survival curves for patients with stage II/III GC stratified by potential confounding factors.***A*, Kaplan-Meier curves for overall survival (OS) in PD-L1^–^ (*left*) or PD-L1^+^ (*right*) patients treated with or without S-1. *B*, Kaplan-Meier curves for OS in patients with low total lymphocyte count (TLC^–^) or high total lymphocyte count (TLC^+^) who did or did not receive S-1 treatment. *C*, subgroup analysis of OS in PD-L1^–^ (*n* = 188) or PD-L1^+^ (*n* = 78) patients (*left*) and TLC^–^ (*n* = 128) or TLC^+^ (*n* = 138) patients (*right*) evaluated for surgery alone and surgery followed by S-1 adjuvant chemotherapy. *D*, positive correlation between inflammation score and *H. pylori* positivity (*left*). Two-tailed *p* values were obtained with the nonparametric Mann-Whitney *U* test. Subgroup analysis of OS in *H. pylori*^–^ (*n* = 131) or *H. pylori*^+^ (*n* = 66) patients evaluated for surgery alone and surgery followed by S-1 adjuvant chemotherapy (*right*). *E*, Kaplan-Meier curves for OS in *H. pylori*^–^ (*left*) or *H. pylori* + (*right*) patients who did or did not receive S-1. *F*, hazards for OS were evaluated for the expression of genes regulated by GC cell-intrinsic signaling in response to proinflammatory cytokines. *G*, Kaplan-Meier curves for OS in ACRG GC patients (*n* = 283) stratified based on tumor *IRF1* (*left*) and *NFKBIA* (*right*) expression levels. Cox proportional hazards model was used to determine the hazard ratio of each mRNA expression level (*C*, *D*, and *F*). Error bars represent 95% confidence intervals. The *p* values were obtained with log-rank test (*A*, *B*, *E*, and *G*). Stratification strategies are shown in Supplemental Fig. S6*A*. ACRG, Asian Cancer Research Group (18).

Unexpectedly, neither overall survival (OS) nor the relapse-free survival (RFS) rate differed between surgery and S-1 adjuvant chemotherapy in PD-L1^−^ patients (Fig. 5*A*; Supplemental Fig. S5*A*). To identify alternative host markers for predicting the effectiveness of anti-PD-L1/PD-1 therapy, we revisited the immune parameters of our cohort. The most remarkable effect was seen when patients were segregated into two groups based on median TLC, where S-1 for TLC^+^ patients demonstrated higher 5-year OS and RFS rates in GC patients than that of surgery alone (5-year OS: 80.7% *versus* 67.2%; hazard ratio: 0.46; 95% confidence interval, 0.23‒0.88; *p* = 0.020; 5-year RFS: 76.5% *versus* 63.3%; hazard ratio: 0.49; 95% confidence interval, 0.26‒0.8.9; *p* = 0.020) (Fig. 5, *B* and *C*; Supplemental Fig. S5, *B–D*). These observations indicate that host could contribute to relapse after S-1 adjuvant chemotherapy in addition to tumor cell-intrinsic 5FU resistance or immunosuppression mechanisms (*i.e.*, activation of PAK–MEK1 pathways or PD-L1 expression). In line with this scenario, *H. pylori* infection, which can recruit T lymphocytes and macrophages to gastric mucosa (47), might confer a potential survival benefit in patients with advanced GC even after gastrectomy (48). However, despite a strong correlation with inflammation scores for gastric mucosa based on the Sydney System (49), the presence of *H. pylori* does not seem to be directly related to TLC (Fig. 5, *D* and *E*; Supplemental Fig. S5, *E* and *F*), suggesting that TLC may reflect an event that is distinct from that of *H. pylori*–induced immunity. Whether *H. pylori*–induced or not, lymphocytes and other tumor-infiltrating immune cells can nonetheless secrete proinflammatory cytokines such as TNFα and IFNγ (50). To examine whether proinflammatory cytokines are associated with survival of patients with GC, we compared the hazard ratios for patient survival outcomes based on expression levels of genes regulated by NFκB and STAT1, which can be activated by TNFα and IFNγ signaling inputs, with an independent cohort (18). Remarkably, expression levels of NFκB/STAT1 targets including *IRF1*, *CD274*, *IRF9*, and *STAT1* are associated with better OS in the cohort (Fig. 5, *F* and *G*). These expression levels are in good agreement with the correlation between TLC and survival outcomes in our cohort. In contrast, other NFκB targets such as *NFKBIA*, *IRF7*, and *IL6* appear to drive opposite functions and outcomes.

**Fig. 6:**
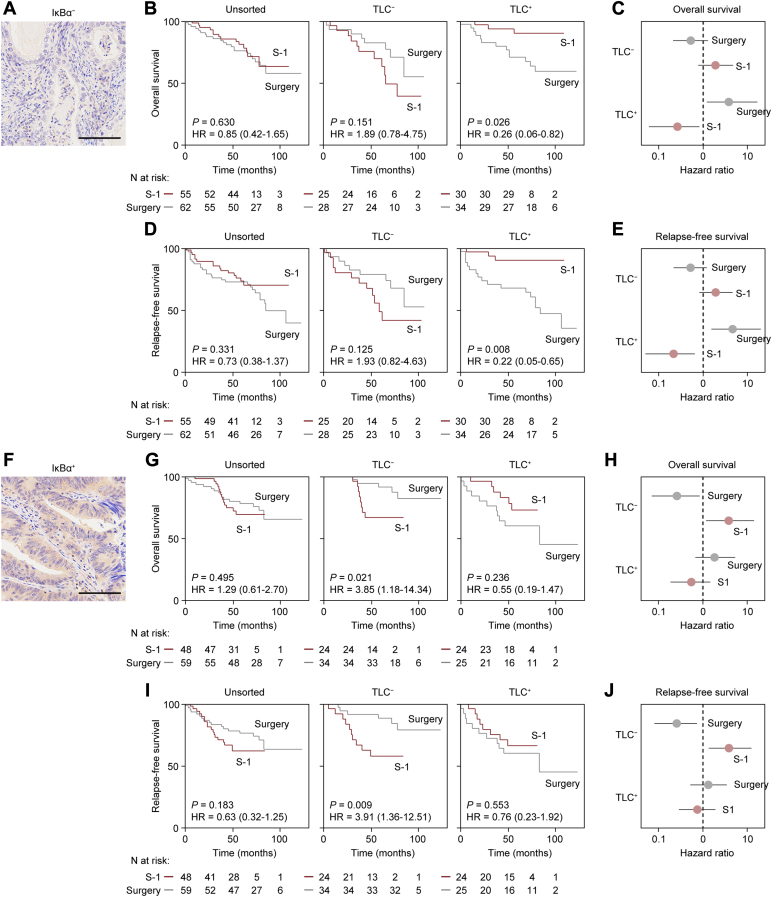
**Survival curves for patients with stage II/III GC stratified by IκBα level and potential confounding factors.***A*, representative immunohistochemical staining in a IκBα^–^ tumor region. *B*, Kaplan-Meier curves for OS in IκBα^–^ patients stratified by treatment (*i.e.*, S-1 and Surgery) divided into TLC^–^ and TLC^+^ groups. *C*, subgroup analysis stratified by interaction of TLC^–^ (*n* = 53) or TLC^+^ (*n* = 64) based on the hazard for OS was evaluated by treatment. *D*, Kaplan-Meier curves for RFS in IκBα^–^ patients stratified by treatment divided into TLC^–^ and TLC^+^ groups. *E*, subgroup analysis stratified by interaction of TLC^–^ (*n* = 53) or TLC^+^ (*n* = 64) based on the hazard for OS was evaluated by treatment. *F*, representative immunohistochemical staining in a IκBα^+^ tumor region. *G*, Kaplan-Meier curves for OS in IκBα^+^ patients stratified by the treatment divided into TLC^–^ and TLC^+^ groups. *H*, subgroup analysis stratified by interaction of TLC^–^ (*n* = 58) or TLC^+^ (*n* = 49) based on the hazard for OS was evaluated by treatment. *I*, Kaplan-Meier curves for RFS in IκBα^+^ patients stratified by treatment divided into TLC^–^ and TLC^+^ groups. *J*, subgroup analysis stratified by interaction of TLC^–^ (*n* = 58) or TLC^+^ (*n* = 49) based on the hazard for RFS was evaluated by treatment. Scale bar, 50 μm (*A* and *F*). Risk for survival was evaluated using Cox proportional hazards models (*C*, *E*, *H* and *J*). Error bars represent 95% confidence intervals. The *p* values were obtained with a log-rank test (*B*, *D*, *G* and *I*).

We next validated the finding that *NFKBIA* expression was associated with poor survival in patients with GC in an immunohistochemical study in our cohort and further examined whether IκBα expression could be a surrogate for low TLC (Supplemental Fig. S6*A*). The number of patients who are TLC^−^ was significantly lower in IκBα^+^ patients than IκBα^−^ patients (Supplemental Fig. S6*B*). By excluding IκBα^−^ patients, S-1 for TLC^+^ patients demonstrated higher 5-year OS and RFS rates than for patients with GC who had surgery alone (5-year OS: 89.9% *versus* 70.6%; hazard ratio: 0.26; 95% confidence interval, 0.06‒0.82; *p* = 0.026; 5-year RFS: 90.0% *versus* 67.7%; hazard ratio: 0.22; 95% confidence interval, 0.05‒0.65; *p* = 0.008) (Fig. 6, *A–D*). Exclusion of IκBα^−^ patients enabled more accurate outcome prediction compared to the method based on only TLC status (Fig. 5*B*; Supplemental Fig. S5*B*). Subgroup analysis showed the potential for substantial reduction in the risk of GC-related death and relapse after S-1 adjuvant chemotherapy in patients who were TLC-IκBα double negative (Fig. 6, *C* and *E*). In addition, S-1 adjuvant chemotherapy provided no survival benefits for IκBα^+^ patients and notably the survival rate was even worser than surgery alone for TLC^−^ patients (Fig. 6, *F*–*J*).

Collectively, TLC predicts a survival benefit for patients with advanced GC who received postoperative S-1 adjuvant chemotherapy. Thus, drug-resistance mechanisms involving NFκB and STAT1 signaling may allow GC cells to respond to proinflammatory cytokines such as TNFα and IFNγ in the patients’ bodies.

## Discussion

The Cancer Genome Atlas and Asian Cancer Research Group established a robust molecular classification method for GCs based on their mutations and gene expression profiles (17, 18). This classification method has been successfully applied to cell line–based compound screening for targeting the EMT subtype (51). In this study, we classified 8 GC cell lines into drug-sensitive and drug-resistant groups based on GI_50_ profiles in addition to comparing matched resistant-parental pairs to determine whether the phenotypic drug response was associated with known GC subtypes. Based on a correlation with vimentin expression, cell lines having dual CIS/ETP sensitivity are considered to be an EMT subtype (*i.e.*, vimentin^+^), into which 15% of gastric tumors are classified (18). An opposite trend was observed in a recent clinical study of platina-based adjuvant chemotherapy in advanced non–small cell lung cancer (52). Although this discrepancy could be explained by the differences between lung and gastric cancers, other possibilities include the fundamental differences between *in vitro* and *in vivo* analysis. As our GSEA shows that transcriptional level of EMT in vimentin-expressing cancer cells was opposed by IFNα response, vimentin-expressing cancer cells could evade cytotoxic IFNα response and exert higher fitness in the patient, particularly in an inflammatory tumor microenvironment. However, the mechanistic link between decreased IFNα responsiveness and CIS resistance remains unclear. A recent study involved in GC organoids revealed that type I IFN induces RNA editing to stabilize mRNA of stearoyl-CoA desaturase that establishes resistance to 5FU and CIS chemotherapy by balancing chemotherapy-induced ER stress (53). Therefore, IFN response should be a key component of CIS resistance in GC.

Dual CIS/ETP sensitivity was also correlated with CNV represented by copy number loss of proximal 4q12–q13, which could be related to *KIT* copy number. Indeed, KIT loss was correlated with CIS/ETP sensitivity in the PRISM cell lines, which share only 1 cell line (MKN1) with our cell line panel. Consistent with these observations, cell lines with dual CIS/ETP sensitivity have decreased expression of DNA repair genes. These cell lines instead possess a highly sensitive caspase activation cascade toward caspase-3/7 that gives a sharp rise in PARP cleavage in the presence of CIS or ETP. Conversely, dual CIS/ETP-resistant cell lines exhibited upregulated expression of DNA repair genes and balanced caspase-3/7 activity whether or not CIS or ETP is present. For therapeutic purposes, nicotinamide phosphoribosyltransferase inhibitors like FK866 and KPT9274 that are reported to selectively target the EMT subtype (51, 54) could be a substitute for CIS, especially when combined with 5FU, for patients with advanced GC. CIS-resistant GC cells could potentially be eliminated by PARP inhibitors such as olaparib, rucaparib, and niraparib based on their PARP dependency, and thus sequential administration of 5FU/CIS and PARP inhibitors warrants evaluation clinically (55).

In contrast to CIS/ETP, 5FU sensitivity had no apparent correlation with known GC subtypes. Our exploratory pathway analysis based on protein expression dynamics after drug administration revealed that 5FU-resistant cell lines, but not cell lines that are resistant to other drugs, have diminished global protein dynamics compared to their sensitive counterparts, with the exception of the MAPK pathway. Subsequent pairwise comparisons of matched 5FU-resistant parental cell line pairs identified PAK-mediated phosphorylation of MEK1 at S298. MEK1 was the only MAPK component that remained active in the presence of 5FU in both 45FU and 74FU cells. In addition to MEK1 phosphorylation, both 5FU-resistant cell lines exhibited increased levels of PD-L1, regardless of the predominant signaling pathways (*i.e.*, G2/M checkpoint activation in 45FU and increased IFN response in 74FU). Interestingly, high levels of PD-L1 are associated with chronic DNA damage in human epithelial cancer cells (56), which could also be mimicked by long-term 5FU culture under which the 45FU and 74FU cell lines arose (15).

Based on the finding that high PD-L1 expression was shared by both 5FU-resistant GC cell lines despite their distinct molecular profiles, we investigated the relationship between PD-L1 expression and prognosis in our cohort of patients with advanced GC. Despite its immunosuppressive function, PD-L1 expression was associated with a lower risk of relapse in patients with advanced GC who underwent gastrectomy followed by S-1 adjuvant chemotherapy. Among the potential confounding factors we investigated, TLC was the most significantly associated with lower risk of GC relapse, suggesting that host immunity could be a major contributor to patient survival. Tumor-infiltrating lymphocytes and other immune effectors secrete cytokines such as TNFα and IFNγ, which can activate NFκB and STAT1 signaling in cancer cells. It has been reported that nuclear expression of RelA is positively correlated with OS rate of patients with GC (57). More recent work showed that IRF1, a downstream target of NFκB and STAT1, opposes multidrug resistance in GC cells (58), although the involvement of NFκB or host lymphocytes has not been adequately explored. In addition to IRF1, we found that gene expression of STAT1, SOCS1, and PD-L1, which can be induced by IFNγ *via* STAT1, was also associated with favorable prognosis for patients with GC. These observations suggest that cytokines produced by tumor-infiltrating immune cells such as TNFα and IFNγ are key components that could be exploited to prevent GC progression. Finally, based on the findings that *NFKBIA* expression was associated with poor survival in patients with GC, we examined whether expression of IκBα encoded by *NFKBIA* could be an indicator of poor outcomes in GC patients in our validation cohort. Strikingly, IκBα stratification could identify TLC^+^ GC patients do not have a survival benefit from postoperative chemotherapy. Continuous turnover of IκBα may signify proinflammatory responses, a hallmark observed in the patient subgroup that benefits from 5FU/platinum therapy alongside immune checkpoint blockade in advanced GC (59).

The present study does have some limitations. First, we used only cell lines for quantitative molecular/phenotypic assays without morphological/localization information. Findings for stress responses and inflammation may require imaging evaluation as well as an assay to clarify the host-tumor response. Second, we did not treat the cell lines with simultaneous and/or pulse drug treatment, which is often applied in daily clinical practice. Third, the studies did not contain a functional immune response or other components of the tumor microenvironment that may be directly affected by the DNA-damaging agents as well as by bidirectional communication with the malignant cells. Finally, the clinical efficacy of potential molecular targeting agents after treatment with DNA-damaging agents remains to be clarified.

Collectively, our multiplatform molecular profiling of GC cell lines, including 5FU-responsive phosphoprotein dynamics, indicates that the mechanisms of 5FU resistance observed in cell line models may be overcome by host immunity. These studies suggest further determination of the best strategies for treatment of advanced CG patients.

## Data Availability

Targeted gene sequencing and RNA-seq data have been deposited in the DNA Data Bank of Japan (DDBJ) *via* the Bioinformation and DDBJ Center under dataset identifier DRA011072 and DRA006585 (http://www.ddbj.nig.ac.jp). RPPA data is available at The University of Texas MD Anderson Cancer Center RPPA Data Repository under data set identifier TCPA00000007 (https://tcpaportal.org). Cohort data for advanced GC patients from the Northern Japan Gastric Cancer Study Consortium are available upon request.

## Supplemental data

This article contains supplemental data (4, 25, 30, 60).

## Conflict of interest

The authors declare no competing interests.
