# Supplementary material for: Targeted Dynamic Phospho-Proteogenomic Analysis of Gastric Cancer Cells Suggests Host Immunity Provides Survival Benefit
Source: Mol Cell Proteomics. 2024 Oct 25;23(12):100870. doi: 10.1016/j.mcpro.2024.100870 (PMC11621936; doi:10.1016/j.mcpro.2024.100870)
Supplement: Supplemental Data [file mmc1.docx]

Supplementary Materials for

**Targeted dynamic phospho-proteogenomic analysis of gastric cancer cells suggests host immunity provides survival benefit**

Kohei Kume *et al.*

*Corresponding author. E-mail: snishizu@iwate-med.ac.jp

**This file includes:**

Supplementary figures S1 to S7

Supplementary materials and methods

Supplementary tables S1 to S2

Supplementary references


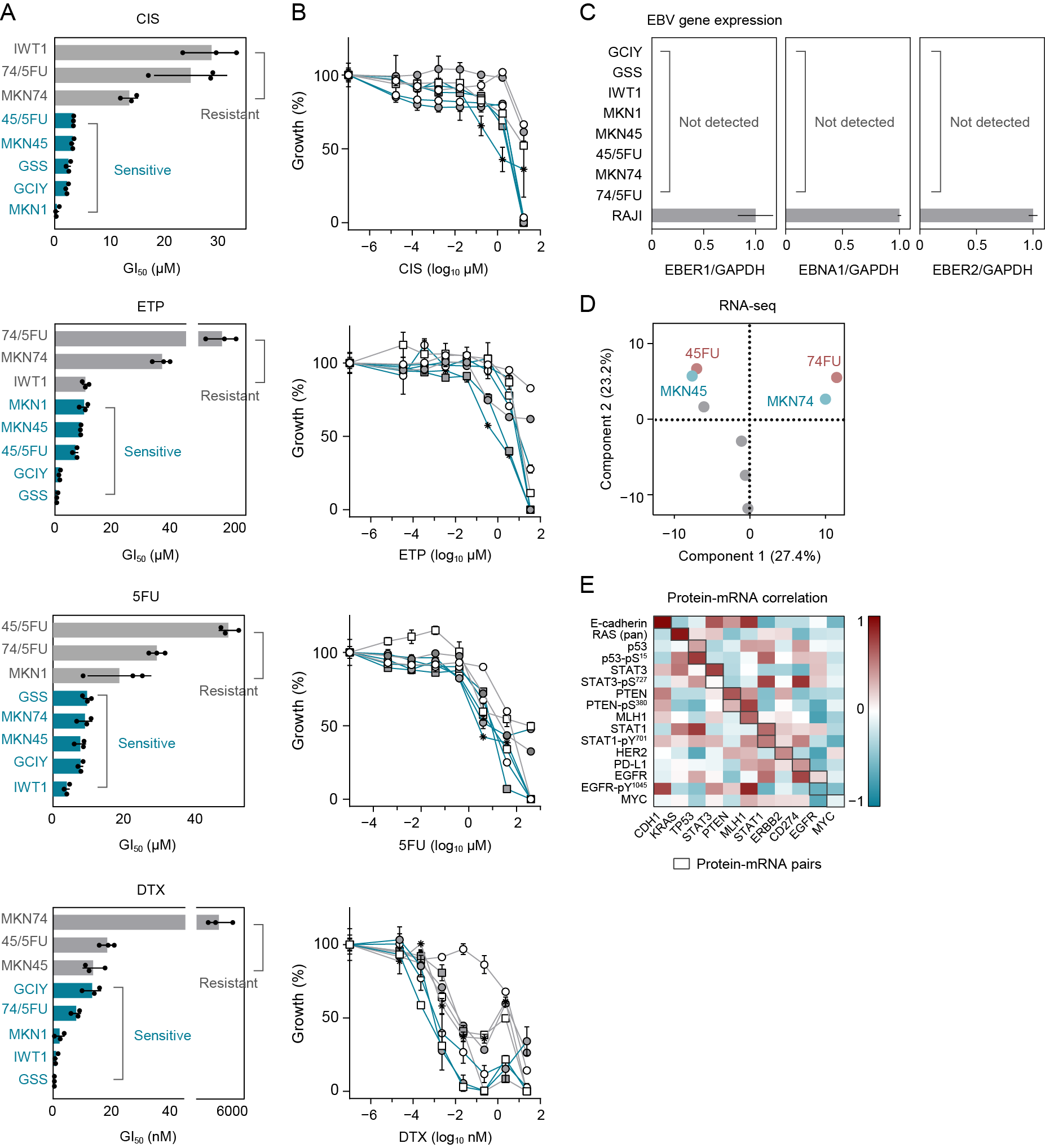


Fig. S1. Phenotypic and proteogenomic profiling of GC cell lines.

(**A**) GI_50_ profiles of GC cell lines to define sensitivity and resistance to each drug. (**B**) Dose-response curves for GI_50_ by 5FU, CIS, ETP, and DTX. (**C**) Quantification of EBV gene expression in GC cell lines. GAPDH was used as an internal control. The EBV-positive cell line RAJI was used as a positive control. (**D**) Principal component analysis of RNA-seq gene expression data. (**E**) Pearson correlations between protein and mRNA levels. Protein-mRNA pairs are highlighted. Experiments were performed in triplicate (A and B) or duplicate (C). Error bars represent s.d. (A), s.e.m. (B) or range (C).


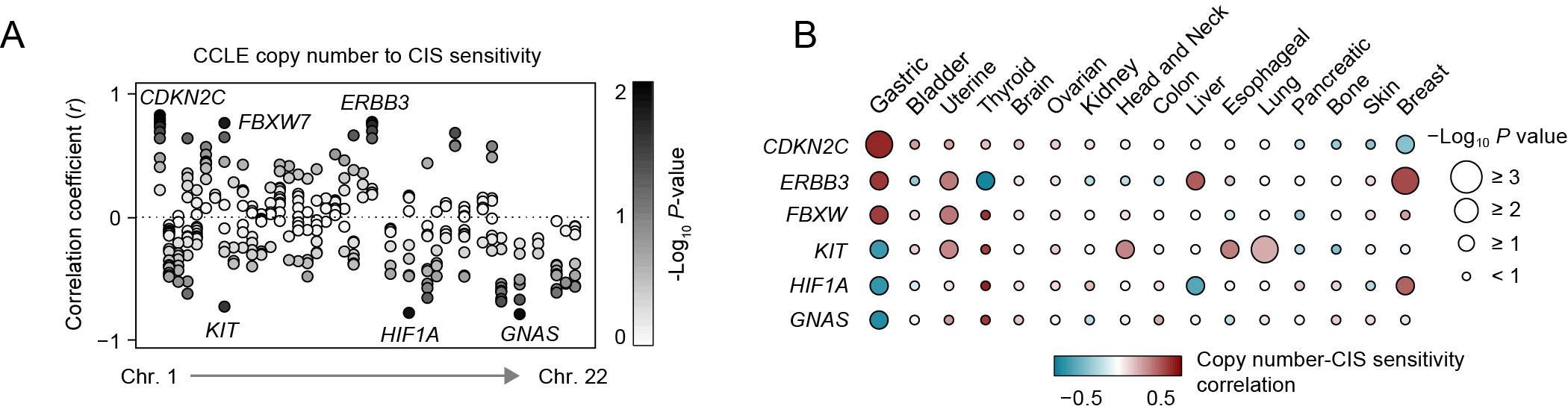


Fig. S2. CIS sensitivity-associated copy number gains and losses in GC cell lines.

(**A**) Correlation coefficient between the gene copy number and CIS sensitivity in GC cell lines. Reciprocals of area under the dose-response curve (AUC) values for CIS were used as CIS sensitivity scores. AUC values were assessed in the PRISM repurposing secondary screen (1). Each dot represents an individual gene ordered by chromosomal position. The top three candidates for both gains and losses are labeled. (**B**) Pan-cancer correlation analysis between the gene copy number and CIS sensitivity. The top three candidates for both gains and losses are shown.


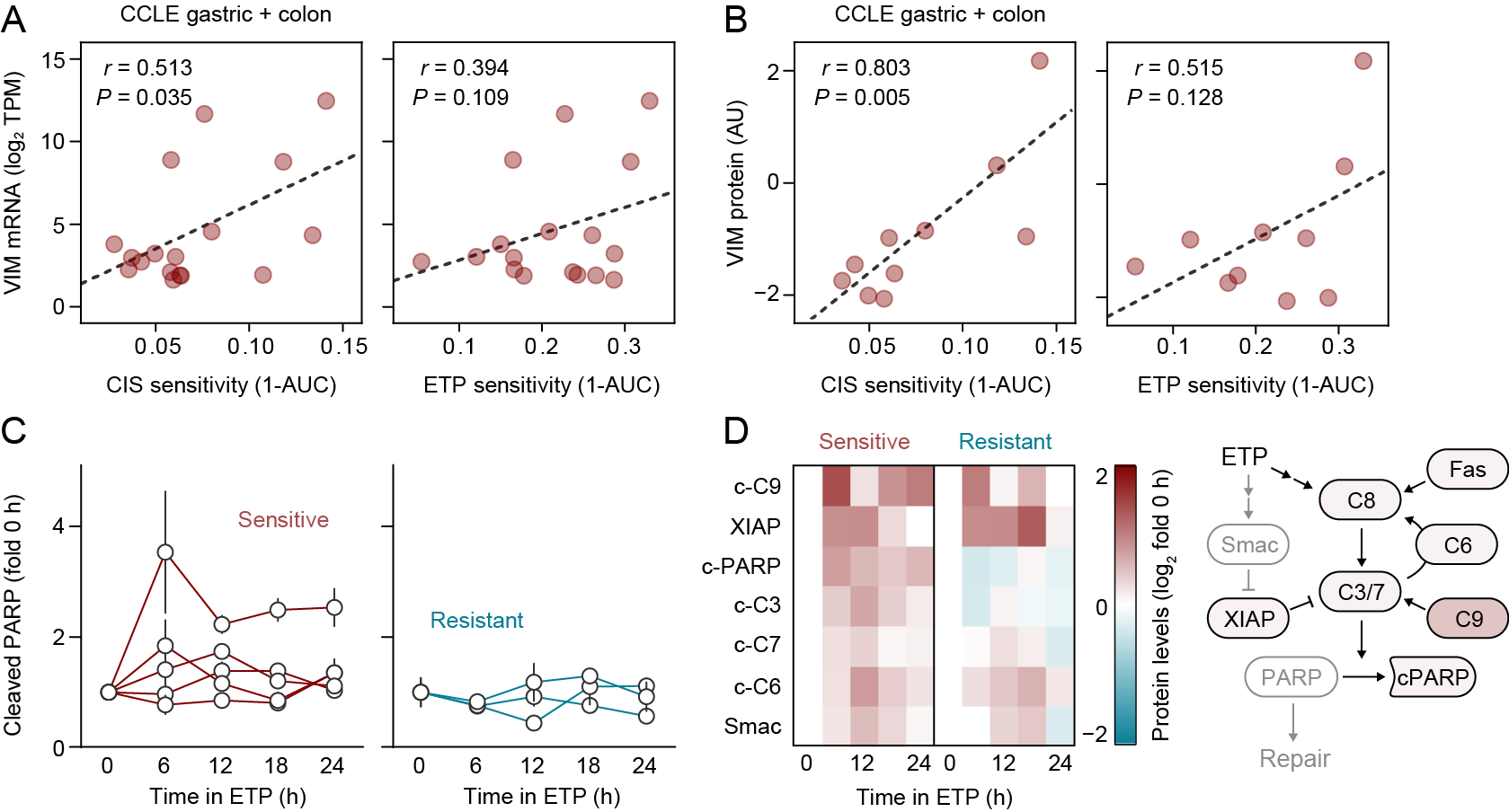


Fig. S3. Dual CIS/ETP sensitivity associated with increased VIM expression and active PARP cleavage.

(**A**) Scatter plots showing correlation between AUC values of CIS or ETP and *VIM* mRNA expression across gastric and colon cancer cell lines. Each dot indicates an individual cell line. (**B**) Scatter plots showing correlation between AUC values of CIS or ETP and vimentin protein levels across gastric and colon cancer cell lines. Each dot indicates an individual cell line. (**C**) Time course RPPA data showing changes in cleaved PARP levels after ETP treatment. Error bars represent s.e.m. (**D**) Caspase-focused RPPA analysis of dual CIS/ETP-sensitive (mean, *n* = 5) and -resistant (mean, *n* = 3) cell lines (left) and a schematic of ETP-activated signaling outcomes in dual CIS/ETP-sensitive cell lines (right). c-PARP, cleaved PARP; c-C3−9, cleaved caspase-3−9.

**
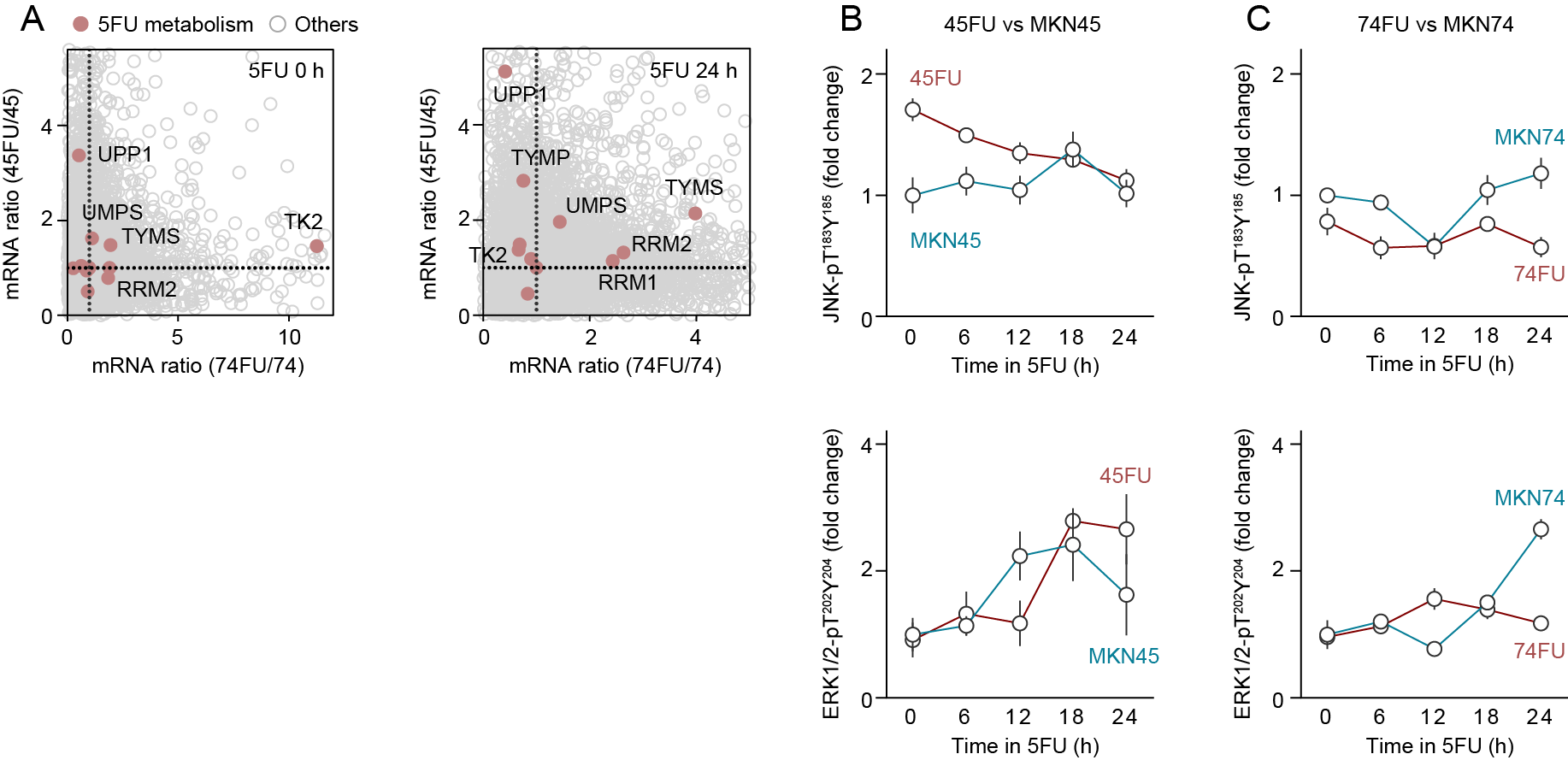
**

Fig. S4. Gene and protein expression profiling of 5FU-resistant cell lines.

(**A**) Gene expression analysis of 5FU-resistant GC cell lines for baseline (left) and 24 h after 5FU treatment (right). mRNA levels in 45FU and 74FU are shown as fold-change compared to their matched parental cell lines and further highlighted for 5FU metabolism pathway genes. (**B**) Temporal changes in JNK-pT^183^/Y^185^, p38-pT^180^/Y^182^, and ERK1/2-pT^202^/Y^204^ levels in MKN45 and 45FU cell lines after 5FU treatment. (**C**) Temporal changes in JNK-pT^183^/Y^185^, p38-pT^180^/Y^182^, and ERK1/2-pT^202^/Y^204^ levels in MKN74 and 74FU cell lines after 5FU treatment. Error bars represent s.e.m. (B and C).

**
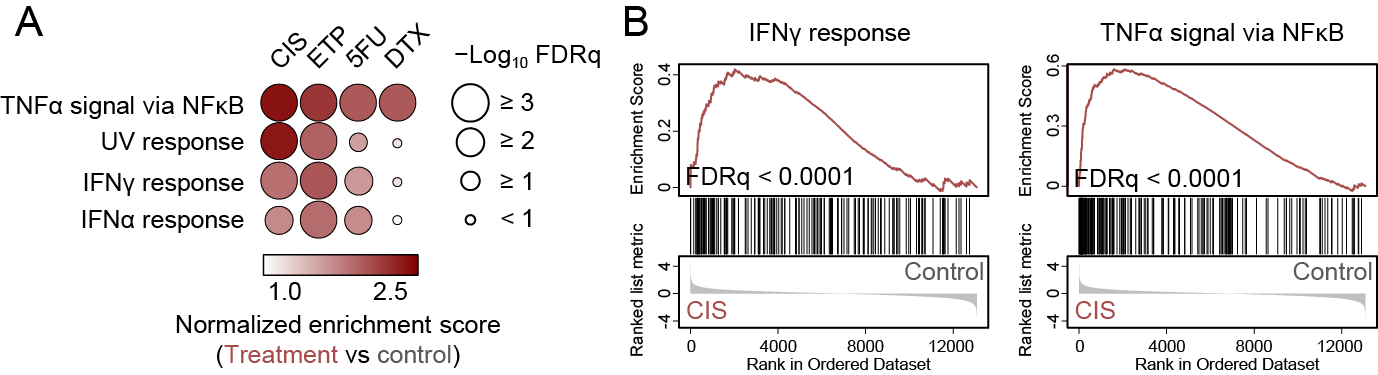
**

Fig. S5. Proinflammatory gene expression induced by chemotherapeutic drugs.

(**A**) Enrichment of “TNFα signaling via NFκB”, “UV response”, “IFNγ response”, and “IFNα response” gene sets in GC cell lines (*n* = 8) treated with the indicated drugs. “UV response” gene set was used as a positive control for DNA-damaging drugs. DTX was used as a non-DNA-damaging drug. (**B**) GSEA curves for “IFNγ response” and “TNFα signaling via NFκB”. FDRq, false discovery rate (*q* value).


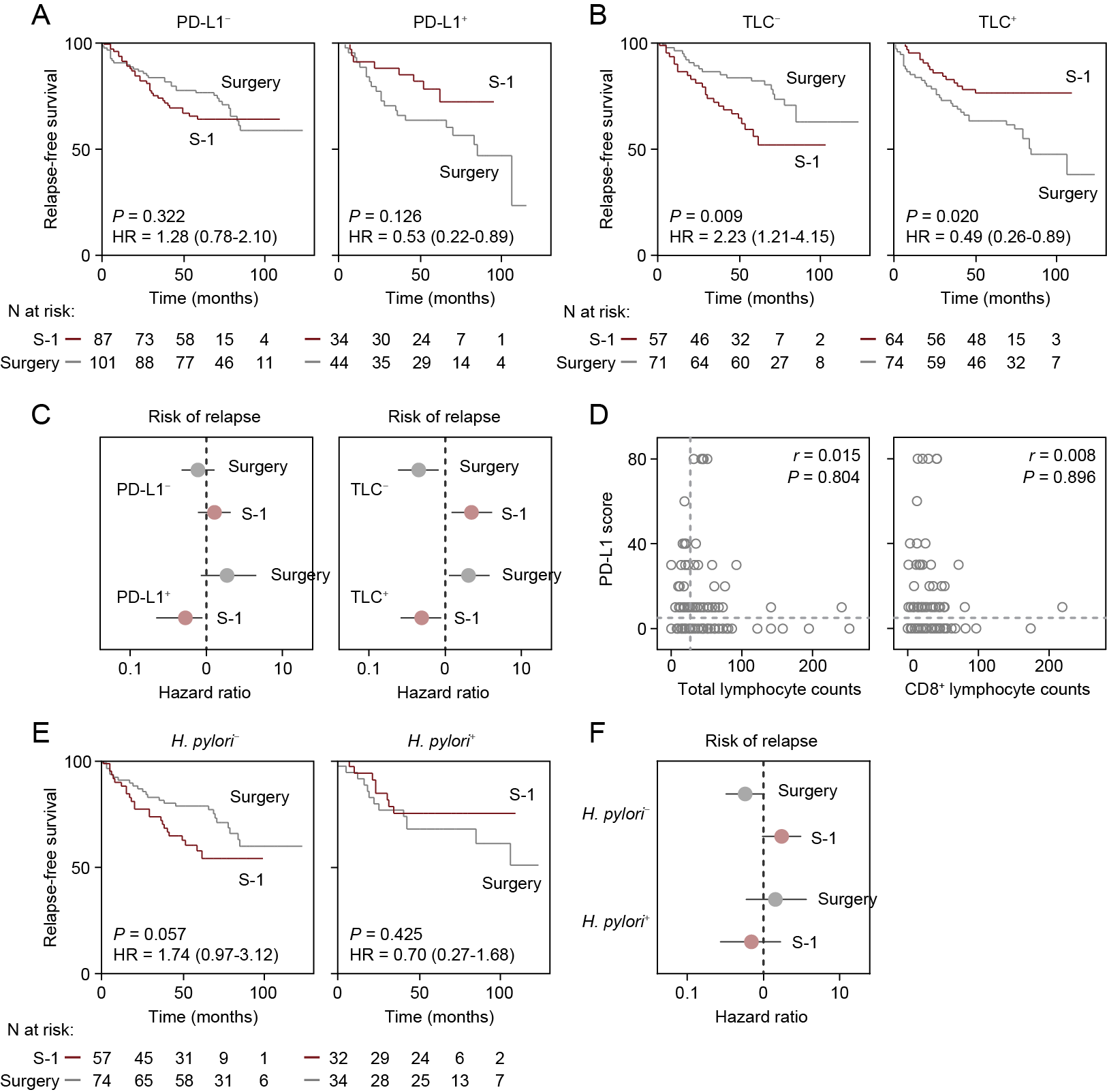


Fig. S6. RFS curves of stage II/III GC patients stratified by potential confounding factors.

(**A**) Kaplan-Meier curves for relapse-free survival (RFS) in PD-L1^–^ (left) or PD-L1^+^ (right) patients by treatment (i.e., S-1 or Surgery). (**B**) Kaplan-Meier curves for RFS in patients with low total lymphocyte count (TLC^–^) or high total lymphocyte count (TLC^+^) by treatment. (**C**) Subgroup analysis stratified by interaction of PD-L1^–^ (n = 188) or PD-L1^+^ (n = 78); and TLC^–^ (n = 128) or TLC^+^ (n = 138) based on the hazard for RFS was evaluated by treatment. (**D**) Scatter plots for PD-L1 score and total lymphocyte count (left) or CD8^+^ lymphocyte count (right). (**E**) Kaplan-Meier curves for RFS in *H. pylori*^–^ (left) or *H. pylori*^+^ (right) patients by treatment. (**F**) Subgroup analysis stratified by interaction of *H. pylori*^–^ (n = 131) or *H. pylori*^+^ (n = 66) based on the hazard for RFS was evaluated by treatment.


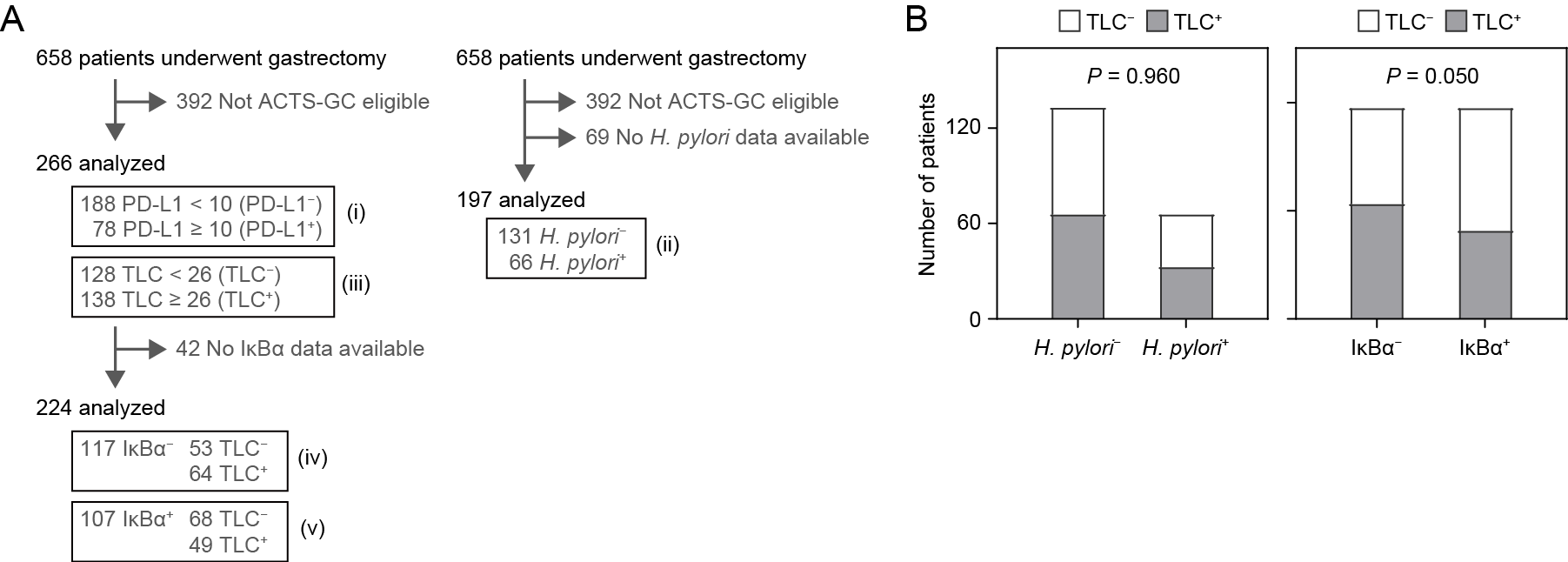


Fig. S7. Association between TLC and IκBα expression in advanced GC.

(**A**) Flowchart for study cohort selection. The number of patients in the stratified groups based on positivity for PD-L1 (i), *H. pylori* (ii), TLC (iii), and combined TLC and IκBα (iv and v) is shown. ACTS-GC, Adjuvant Chemotherapy Trial of S-1 for Gastric Cancer(2). (**B**) Chi-square exact test for TLC and *H. pylori* positivity (left) and TLC and IκBα positivity (right). Risk of relapse was estimated using Cox proportional hazards models (C and F). Error bars represent 95% confidence intervals. The *P* values were obtained with log-rank test (A, B, and E).

Supplementary materials and methods

**Drugs**

5FU and DTX were purchased from Kyowa-Hakko Bio (Tokyo, Japan), and Sanofi KK (Tokyo, Japan), respectively. CIS and ETP were purchased from Nippon Kayaku (Tokyo, Japan).

**Growth suppression assay**

Cells were plated in a 96-well plate at 1.0 to 4.0 ´ 10^4^ cells/well. Cell viability was determined using CCK-8 (Dojindo, Kumamoto, Japan) and a TriStar LB 941 microplate reader (Berthold Technologies, Bad Wildbad, Germany). GI_50_ values were calculated using GraphPad Prism software version 7.0 (GraphPad software Inc., San Diego, CA, USA).

**Targeted gene sequencing**

Genotyping using the Ion AmpliSeq Pharmacogenomics Research Panel was conducted according to the manufacturer’s instructions using the Ion Torrent platform (Thermo Fisher Scientific, Waltham, MA, USA). This panel analyzes genetic variants including SNPs, insertion/deletions, and CNVs that are associated with 40 drug metabolizing enzymes. Gene mutation profiling was also performed using the Ion AmpliSeq Comprehensive Cancer Panel that covers nearly all coding regions of 409 genes that are known to be related to cancer (Thermo Fisher Scientific).

**RNA-seq**

Total RNA was extracted using an Rneasy Kit (Qiagen), according to the manufacturer’s instructions. RNA-Seq libraries were constructed using TruSeq Stranded mRNA HT Sample Prep Kit (Illumina Inc, San Diego, CA, USA). Sequencing was carried out on the Illumina HiSeq3000 platform with a 36-base-single end run. Quality control of RNA-seq reads was performed using FastQC version 0.11.5. Raw sequence reads were mapped to the UCSC genome for human (hg19) using HISAT2 version 2.1.0, without novel splice variant discovery. Fragments per kilobase of exon per million (FPKM) mapped reads were calculated from the mapped reads using Ballgown version 2.6.0. FPKM data was later used in gene set enrichment analysis (GSEA) performed with GSEA Software (version 4.1.0) and WebGestalt (3).

**qRT-PCR**

Total RNA was extracted from GC cell lines using an RNeasy kit (QIAGEN). cDNA was synthesized from 500 ng total RNA in a 10 μl reaction volume using PrimeScript RT Master Mix (TaKaRa Bio, Otsu, Japan). Quantitative RT-PCR (qRT-PCR) was performed using the LightCycler Nano System (Roche, Mannheim, Germany). Primer sequences are shown in Table S4.

**Immunohistochemistry**

A tissue microarray (TMA) including 658 GC specimens was made for immunohistochemistry (IHC) staining. The primary antibodies used were: rabbit anti-IκBα polyclonal antibody (E-AB-70089, Elabscience, Houston, TX, USA), mouse anti-PD-L1 monoclonal antibody (22C3, Dako, Santa Clara, CA, USA), and CD4/CD8 (4B12/SP16; Biocare Medical, Concord, Pacheco, CA, USA). For IκBα scoring, cases in which >30% of all tumor cells were stained were defined as positive. The staining evaluation was focused on the cytoplasm in the epithelial component. For staining evaluation, the MKN1 cell line was used as a positive control, and cases having equal or greater positivity relative to the positive control were considered positive. All scoring was performed by an independent pathologist (A.Y-A.), who was blinded to the clinical outcomes. The scoring algorithms and staining evaluation for PD-L1 and CD4/CD8 have been described previously (4).

Table S1. Drug concentrations for the time course RPPA analysis.

| Drug | Low | Middle | High |
| --- | --- | --- | --- |
| 5FU | 0.38 µM | 3.8 µM | 38 µM |
| CIS | 0.17 µM | 1.7 µM | 17 µM |
| ETP | 0.34 µM | 3.4 µM | 34 µM |
| DTX | 0.23 µM | 2.3 µM | 23 µM |

Table S2. Primary antibodies used for RPPA.

| Category | Target | Vendor | Catalog no. | Dilution |
| --- | --- | --- | --- | --- |
| Phospho | AKT-pS^473^ | Cell Signaling | 9271 | 1:100 |
| Phospho | A-Raf-pS^299^ | Cell Signaling | 4431 | 1:50 |
| Phospho | ASK1-pS^83^ | Cell Signaling | 3761 | 1:50 |
| Phospho | ATF2-pT^71^ | Santa Cruz | sc-8398 | 1:100 |
| Phospho | ATF2-pT^71^ | Cell Signaling | 9221 | 1:100 |
| Phospho | ATM-pS^1981^ | Cell Signaling | 5883 | 1:100 |
| Phospho | ATR-pS^428^ | Cell Signaling | 2853 | 1:100 |
| Phospho | Aurora A-pT^288^/B-pT^232^/C-pT^198^ | Cell Signaling | 2914 | 1:100 |
| Phospho | BAD-pS^112^ | Cell Signaling | 9291 | 1:200 |
| Phospho | Bcl2-pS^70^ | Cell Signaling | 2827 | 1:100 |
| Phospho | β-arrestin1-pS^412^ | Cell Signaling | 2416 | 1:100 |
| Phospho | β-catenin-pT^41^/S^45^ | Cell Signaling | 9565 | 1:100 |
| Phospho | Chk1-pS^345^ | Cell Signaling | 2341 | 1:100 |
| Phospho | EGFR-pY^1045^ | Cell Signaling | 2237 | 1:100 |
| Phospho | ELK1-pS^383^ | Cell Signaling | 9181 | 1:200 |
| Phospho | ERK1/2-pT^202^/Y^204^ | Cell Signaling | 9101 | 1:500 |
| Phospho | FADD-pS^194^ | Cell Signaling | 2781 | 1:100 |
| Phospho | Histone H3-pS^28^ | Sigma-Aldrich | 07-145 | 1:500-1000 |
| Phospho | HSP90-pT^5/7^ | Cell Signaling | 3488 | 1:100-500 |
| Phospho | IR-pY^1150/51^/IGF1R-pY^1135/36^ | Cell Signaling | 3024 | 1:500 |
| Phospho | IκBα-pS^32/36^ | Cell Signaling | 9246 | 1:100 |
| Phospho | Jak2-pY^1007/1008^ | Cell Signaling | 3771 | 1:200 |
| Phospho | JNK/SAPK-pT^183^/Y^185^ | Cell Signaling | 9251 | 1:100 |
| Phospho | LKB1-pS^428^ | Cell Signaling | 3051 | 1:100 |
| Phospho | MEK1/2-pS^217/221^ | Cell Signaling | 9121 | 1:200 |
| Phospho | MEK1-pS^298^ | Cell Signaling | 9128 | 1:2000 |
| Phospho | Mst1-pT^183^/Mst2-pT^180^ | Cell Signaling | 3681 | 1:100 |
| Phospho | mTOR-pS^448^ | Cell Signaling | 2971 | 1:100 |
| Phospho | NPM-pT^199^ | Cell Signaling | 3541 | 1:100 |
| Phospho | p27-pT^187^ | Sigma-Aldrich | 71-7700 | 1:200 |
| Phospho | p38-pT^180^/T^182^ | Cell Signaling | 9211 | 1:100 |
| Phospho | p53-pS^15^ | Cell Signaling | 9284 | 1:1000 |
| Phospho | p70 S6K-pS^371^ | Cell Signaling | 9208 | 1:50 |
| Phospho | p70 S6K-pT^389^ | Cell Signaling | 9234 | 1:500 |
| Phospho | p90 RSK-pT^359^/S^363^ | Cell Signaling | 9344 | 1:200 |
| Phospho | p90 RSK-pS^380^ | Cell Signaling | 9341 | 1:200 |
| Phospho | PAK1-pS^199/204^/PAK2-pS^192/197^ | Cell Signaling | 2605 | 1:100 |
| Phospho | PAK1-pT^423^/PAK2-pT^402^ | Cell Signaling | 2601 | 1:100 |
| Phospho | PDK1-pS^241^ | Cell Signaling | 3061 | 1:200 |
| Phospho | PI3K p85-pY^458^/p55-pY^199^ | Cell Signaling | 4228 | 1:100 |
| Phospho | PKCβII-pS^660^ (pan) | Cell Signaling | 9371 | 1:100 |
| Phospho | PP2A-B | Cell Signaling | 4953 | 1:1000 |
| Phospho | PRK1-pT^774^/PRK2-pT^816^ | Cell Signaling | 2611 | 1:100 |
| Phospho | PTEN-pS^380^ | Cell Signaling | 9551 | 1:500 |
| Phospho | Raf1/C-Raf-pS^259^ | Cell Signaling | 9421 | 1:100 |
| Phospho | Raf1/C-Raf-pS^338^ | Cell Signaling | 9427 | 1:200 |
| Phospho | Ras (pan) | Santa Cruz | sc-166691 | 1:100-200 |
| Phospho | Rb-pS^780^ | Cell Signaling | 3590 | 1:2000 |
| Phospho | RSK3-pT^356^/S^360^ | Cell Signaling | 9348 | 1:500 |
| Phospho | S6-pS^235/236^ | Cell Signaling | 4858 | 1:200 |
| Phospho | S6-pS^240/244^ | Cell Signaling | 5364 | 1:200 |
| Phospho | SEK1/MKK4-pS^80^ | Cell Signaling | 9155 | 1:200 |
| Phospho | SMAD2-pS^465/467^ | Cell Signaling | 3101 | 1:200 |
| Phospho | STAT1-pY^701^ | Cell Signaling | 9171 | 1:500 |
| Phospho | STAT3-pS^727^ | Cell Signaling | 9134 | 1:100 |
| Phospho | TAB2-S^372^ | Cell Signaling | 8155 | 1:100 |
| Phospho | YAP-pS^127^ | Cell Signaling | 13008 | 1:200 |
| Cleaved | Caspase-3, cleaved | Cell Signaling | 9661 | 1:200 |
| Cleaved | Caspase-6, cleaved | Cell Signaling | 9761 | 1:50-100 |
| Cleaved | Caspase-7, cleaved | Cell Signaling | 9491 | 1:100 |
| Cleaved | Caspase-9, cleaved | Cell Signaling | 9505 | 1:100 |
| Cleaved | Caspase-9, cleaved | Cell Signaling | 9501 | 1:100 |
| Cleaved | PARP, cleaved | Cell Signaling | 9541 | 1:100 |
| Total | Abl SH2 domain | Sigma-Aldrich | 06-465 | 1:500 |
| Total | ATG7 | Cell Signaling | 2631 | 1:100 |
| Total | Aurora A | Santa Cruz | sc-373856 | 1:100 |
| Total | BAX | Cell Signaling | 2772 | 1:200 |
| Total | Bcl-xL | Cell Signaling | 2762 | 1:200 |
| Total | β-actin | Santa Cruz | sc-47778 | 1:100 |
| Total | β-catenin | Cell Signaling | 9582 | 1:100 |
| Total | BIM | Cell Signaling | 2933 | 1:500 |
| Total | Caspase-2 | BD | 611022 | 1:10 |
| Total | Caspase-3 | Cell Signaling | 9662 | 1:100 |
| Total | Caspase-7 | Cell Signaling | 9492 | 1:100 |
| Total | Caspase-9 | Cell Signaling | 9502 | 1:500 |
| Total | CD13 | Santa Cruz | sc-13536 | 1:100 |
| Total | CD133 | Cell Signaling | 9196 | 1:100 |
| Total | CDK1 | BD | 61037 | 1:200 |
| Total | CDK2 | BD | 61045 | 1:200 |
| Total | CDK4 | BD | 61047 | 1:200 |
| Total | c-kit/KIT | Santa Cruz | sc-365504 | 1:500 |
| Total | c-kit/KIT | Thermo | 34-8800 | 1:500 |
| Total | Cox-2 | BD | 610203 | 1:200 |
| Total | CXCR4 | Santa Cruz | sc-53534 | 1:100 |
| Total | Cyclin A | BD | 611268 | 1:50 |
| Total | Cyclin A2 | Cell Signaling | 4656 | 1:50 |
| Total | Cyclin B | BD | 610219 | 1:500 |
| Total | Cyclin B1 | Cell Signaling | 4135 | 1:200 |
| Total | Cyclin D1 | BD | 554180 | 1:100 |
| Total | Cyclin D3 | Cell Signaling | 2936 | 1:500 |
| Total | Cyclin E | Cell Signaling | 4129 | 1:500 |
| Total | Cytokeratin 19 | Santa Cruz | sc-37126 | 1:1000 |
| Total | Cytokeratin 8 | Santa Cruz | sc-8020 | 1:1000 |
| Total | E-cadherin | Cell Signaling | 3195 | 1:50 |
| Total | EGFR | Cell Signaling | 2232 | 1:100 |
| Total | EpCAM | Santa Cruz | sc-71057 | 1:100 |
| Total | ErbB2/HER2 | Cell Signaling | 2242 | 1:100 |
| Total | ERK1/2 | Cell Signaling | 9102 | 1:100 |
| Total | GAPDH | Cell Signaling | 2118 | 1:500 |
| Total | GSK3α | Cell Signaling | 9337 | 1:100 |
| Total | GSK3α/β | Santa Cruz | sc-7291 | 1:100 |
| Total | GSK3β | Cell Signaling | 9332 | 1:100 |
| Total | HDAC1 | Cell Signaling | 2062 | 1:200 |
| Total | HDAC3 | Cell Signaling | 2632 | 1:1000 |
| Total | HDAC4 | Cell Signaling | 2072 | 1:100 |
| Total | HDAC6 | Santa Cruz | sc-11420 | 1:2000 |
| Total | Histone H3 | Abcam | ab1791 | 1:10000 |
| Total | HMGB1 | Cell Signaling | 6893 | 1:200 |
| Total | HSP70 | StressGen | SPA-810 | 1:200 |
| Total | HSP90 | Cell Signaling | 4875 | 1:50-100 |
| Total | IGF1R β | Cell Signaling | 3027 | 1:1000 |
| Total | IκBα | Cell Signaling | 9242 | 1:500-1000 |
| Total | JNK/SAPK | BD | 610627 | 1:100 |
| Total | JNK/SAPK | Cell Signaling | 9252 | 1:500 |
| Total | Ki67 | Cell Signaling | 4719 | 1:500 |
| Total | LC3B | Cell Signaling | 2775 | 1:100 |
| Total | MDR1 | Sigma-Aldrich | P7965 | 1:100 |
| Total | MEK1/2 | Cell Signaling | 9122 | 1:1000 |
| Total | MGMT | Cell Signaling | 2739 | 1:100 |
| Total | MLH1 | Cell Signaling | 3515 | 1:500 |
| Total | mTOR | Cell Signaling | 2972 | 1:200 |
| Total | MYD88 | Cell Signaling | 4283 | 1:500 |
| Total | MYC | Santa Cruz | sc-40 | 1:100 |
| Total | NANOG | Cell Signaling | 3580 | 1:1000 |
| Total | NANOG | Cell Signaling | 4903 | 1:100 |
| Total | N-cadherin | Cell Signaling | 4061 | 1:100 |
| Total | NFκB/RelA | Cell Signaling | 3034 | 1:100 |
| Total | Notch1 | Santa Cruz | sc-376403 | 1:100 |
| Total | NRF2 | Abcam | ab62352 | 1:200 |
| Total | p21 | Santa Cruz | sc-6246 | 1:50-100 |
| Total | p21 | Cell Signaling | 2946 | 1:200 |
| Total | p27 | BD | 610242 | 1:100 |
| Total | p38 | Cell Signaling | 9212 | 1:200 |
| Total | p53 | Invitrogen | AHO0152 | 1:200 |
| Total | p53 | Thermo | MS-187 | 1:100 |
| Total | p70/p85 S6K | Cell Signaling | 2708 | 1:100-1000 |
| Total | p70/p85 S6K | Cell Signaling | 9202 | 1:100-1000 |
| Total | PCAF | Cell Signaling | 3378 | 1:500 |
| Total | PD-L1 | Cell Signaling | 13684 | 1:5000 |
| Total | PI3K p85 | Cell Signaling | 4292 | 1:500 |
| Total | PIAS1 | Cell Signaling | 3550 | 1:100 |
| Total | PKCα | Upstate | 05-154 | 1:100-200 |
| Total | PP2A-B | Cell Signaling | 4953 | 1:1000 |
| Total | PTEN | Cell Signaling | 9552 | 1:50-100 |
| Total | PUMA | Cell Signaling | 4976 | 1:200 |
| Total | Ras (pan) | Santa Cruz | sc-166691 | 1:100-200 |
| Total | RSK2 | Santa Cruz | sc-9986 | 1:200 |
| Total | Smac/Diablo | Cell Signaling | 2954 | 1:10000 |
| Total | SOX2 | Cell Signaling | 2748 | 1:100 |
| Total | SSEA4 | Cell Signaling | 4755 | 1:100 |
| Total | SRC | Santa Cruz | sc-18 | 1:200 |
| Total | STAT1 | Cell Signaling | 9172 | 1:100-200 |
| Total | STAT3 | Cell Signaling | 9132 | 1:500 |
| Total | SUMO1 | Cell Signaling | 4930 | 1:200 |
| Total | Survivin | Cell Signaling | 2808 | 1:500 |
| Total | TRA-1-60 | Cell Signaling | 4746 | 1:100-200 |
| Total | Tubulin | Cell Signaling | 2148 | 1:100-1000 |
| Total | TWIST | Santa Cruz | sc-81417 | 1:100 |
| Total | UBC3/Cdc34 | Cell Signaling | 4997 | 1:100 |
| Total | Ubiquitin | Cell Signaling | 3936 | 1:50 |
| Total | VDR | Santa Cruz | sc-13133 | 1:100 |
| Total | VHL | Cell Signaling | 2738 | 1:1000 |
| Total | Wnt5a/b | Cell Signaling | 2530 | 1:100 |
| Total | XIAP | Cell Signaling | 2042 | 1:100 |
| Total | ZO-1 | Cell Signaling | 5406 | 1:100 |
| Others | pY | Sigma-Aldrich | 05-321 | 1:1000 |
| Others | pY | Cell Signaling | 9411 | 1:1500 |
| Others | Proteasome 20S C2 | abcam | ab3325 | 1:500 |
| Others | Ubiquitin (K48 linkage) | Cell Signaling | 4289 | 1:100 |

Table S3. Gastric and colon cancer cell lines in the PRISM repurposing primary screen.

| Name | Primary disease | Depmap ID. |
| --- | --- | --- |
| 2313287 | Gastric cancer | ACH-000948 |
| AGS | Gastric cancer | ACH-000880 |
| LMSU | Gastric cancer | ACH-000255 |
| MKN1 | Gastric cancer | ACH-000351 |
| MKN7 | Gastric cancer | ACH-000678 |
| NUGC3 | Gastric cancer | ACH-000911 |
| SH10TC | Gastric cancer | ACH-000764 |
| SNU668 | Gastric cancer | ACH-000344 |
| GP2D | Colon/Colorectal cancer | ACH-000982 |
| HCT116 | Colon/Colorectal cancer | ACH-000971 |
| HCT15 | Colon/Colorectal cancer | ACH-000997 |
| RCM1 | Colon/Colorectal cancer | ACH-000565 |
| RKO | Colon/Colorectal cancer | ACH-000943 |
| SNU81 | Colon/Colorectal cancer | ACH-000991 |
| SNUC2A | Colon/Colorectal cancer | ACH-000967 |
| SNUC4 | Colon/Colorectal cancer | ACH-000959 |
| SW48 | Colon/Colorectal cancer | ACH-000958 |

Table S4. RT-PCR primers.

| Gene | Forward | Reverse |
| --- | --- | --- |
| EBER1 | AGGACCTACGCTGCCCTAGA | GGGAAGACAACCACAGACAC |
| EBER2 | AGGACAGCCGTTGCCCTAGTGG | GCAAATGCTCTAGGCGGGAAG |
| EBNA1 | CCTCCCTGGTTTCCACCTAT | TCCTCACCCTCATCTCCATC |
| GAPDH | AATCCCATCACCATCTTCCA | TGGACTCCACGACGTACTCA |

Supplementary references

1. Corsello, S. M., Nagari, R. T., Spangler, R. D., Rossen, J., Kocak, M., Bryan, J. G., Humeidi, R., Peck, D., Wu, X., Tang, A. A., Wang, V. M., Bender, S. A., Lemire, E., Narayan, R., Montgomery, P., Ben-David, U., Garvie, C. W., Chen, Y., Rees, M. G., Lyons, N. J., McFarland, J. M., Wong, B. T., Wang, L., Dumont, N., O'Hearn, P. J., Stefan, E., Doench, J. G., Harrington, C. N., Greulich, H., Meyerson, M., Vazquez, F., Subramanian, A., Roth, J. A., Bittker, J. A., Boehm, J. S., Mader, C. C., Tsherniak, A., and Golub, T. R. (2020) Discovering the anti-cancer potential of non-oncology drugs by systematic viability profiling. *Nat Cancer* 1, 235-248

2. Sakuramoto, S., Sasako, M., Yamaguchi, T., Kinoshita, T., Fujii, M., Nashimoto, A., Furukawa, H., Nakajima, T., Ohashi, Y., Imamura, H., Higashino, M., Yamamura, Y., Kurita, A., Arai, K., and Group, A.-G. (2007) Adjuvant chemotherapy for gastric cancer with S-1, an oral fluoropyrimidine. *N Engl J Med* 357, 1810-1820

3. Liao, Y., Wang, J., Jaehnig, E. J., Shi, Z., and Zhang, B. (2019) WebGestalt 2019: gene set analysis toolkit with revamped UIs and APIs. *Nucleic Acids Res* 47, W199-W205

4. Koizumi, Y., Ahmad, S., Ikeda, M., Yashima-Abo, A., Espina, G., Sugimoto, R., Sugai, T., Iwaya, T., Tamura, G., and Koeda, K. (2022) Helicobacter pylori modulated host immunity in gastric cancer patients with S-1 adjuvant chemotherapy. *JNCI: Journal of the National Cancer Institute*
